# Adaptation of iCLIP to plants determines the binding landscape of the clock-regulated RNA-binding protein *At*GRP7

**DOI:** 10.1186/s13059-017-1332-x

**Published:** 2017-10-31

**Authors:** Katja Meyer, Tino Köster, Christine Nolte, Claus Weinholdt, Martin Lewinski, Ivo Grosse, Dorothee Staiger

**Affiliations:** 1 RNA Biology and Molecular Physiology, Faculty of Biology, Bielefeld University, Bielefeld, Germany; 2 Institute of Computer Science, Martin Luther University Halle-Wittenberg, Halle, Germany; 3 German Centre for Integrative Biodiversity Research (iDiv) Halle-Jena-Leipzig, Leipzig, Germany

**Keywords:** Circadian rhythm, Individual nucleotide resolution crosslinking and immunoprecipitation (iCLIP), RNA immunoprecipitation (RIP), RNA-binding protein

## Abstract

**Background:**

Functions for RNA-binding proteins in orchestrating plant development and environmental responses are well established. However, the lack of a genome-wide view of their in vivo binding targets and binding landscapes represents a gap in understanding the mode of action of plant RNA-binding proteins. Here, we adapt individual nucleotide resolution crosslinking and immunoprecipitation (iCLIP) genome-wide to determine the binding repertoire of the circadian clock-regulated *Arabidopsis thaliana* glycine-rich RNA-binding protein *At*GRP7.

**Results:**

iCLIP identifies 858 transcripts with significantly enriched crosslink sites in plants expressing *At*GRP7-GFP that are absent in plants expressing an RNA-binding-dead *At*GRP7 variant or GFP alone. To independently validate the targets, we performed RNA immunoprecipitation (RIP)-sequencing of *At*GRP7-GFP plants subjected to formaldehyde fixation. Of the iCLIP targets, 452 were also identified by RIP-seq and represent a set of high-confidence binders. *At*GRP7 can bind to all transcript regions, with a preference for 3′ untranslated regions. In the vicinity of crosslink sites, U/C-rich motifs are overrepresented. Cross-referencing the targets against transcriptome changes in *At*GRP7 loss-of-function mutants or *At*GRP7-overexpressing plants reveals a predominantly negative effect of *At*GRP7 on its targets. In particular, elevated *At*GRP7 levels lead to damping of circadian oscillations of transcripts, including *DORMANCY/AUXIN ASSOCIATED FAMILY PROTEIN2* and *CCR-LIKE*. Furthermore, several targets show changes in alternative splicing or polyadenylation in response to altered *At*GRP7 levels.

**Conclusions:**

We have established iCLIP for plants to identify target transcripts of the RNA-binding protein *At*GRP7. This paves the way to investigate the dynamics of posttranscriptional networks in response to exogenous and endogenous cues.

**Electronic supplementary material:**

The online version of this article (doi:10.1186/s13059-017-1332-x) contains supplementary material, which is available to authorized users.

## Background

RNA-binding proteins (RBPs) regulate RNA processing steps from synthesis to decay, including pre-mRNA splicing, transport, 3′ end formation, translation, and degradation. This regulation at the RNA level represents an important checkpoint to extensively modulate gene expression once transcription has been initiated. *Arabidopsis thaliana* harbors 197 proteins with an RNA recognition motif (RRM), the most frequent type of RNA-binding domain [1]. The complete binding repertoire of any of these RBPs is virtually unknown.

To date, global mapping of in vivo RNA–protein interactions is performed by immunopurification of RNA-binding proteins using antibodies against the native protein or an epitope, and cataloguing the associated RNAs by RNA-seq. In higher plants, RBPs were immunoprecipitated from lysates of purified maize chloroplasts under native conditions and RNAs were identified by microarrays [2]. To preserve the physiological RNA–protein interactions, RNA and bound proteins are often crosslinked in vivo. In conventional RNA immunoprecipitation (RIP) techniques, formaldehyde is used for crosslinking. RIP and subsequent identification of bound transcripts by reverse transcription (RT)-PCR has been used to confirm candidate in vivo targets of plant RBPs [3, 4]. In a first RIP-seq analysis in *Arabidopsis*, more than 4000 targets of the serine/arginine rich (SR)-like protein SR45 were identified by RNA immunoprecipitation, followed by high-throughput sequencing [5].

While RIP is useful to identify in vivo target transcripts, it does not provide immediate information about the binding motifs on the RNAs. To overcome this drawback, more recently developed crosslinking and immunoprecipitation (CLIP) techniques rely on UV-induced covalent bonds between RBPs and their target RNAs, providing information on the site of interaction [6]. For CLIP, adapters are attached to both the 5′ and 3′ ends of the RNAs co-precipitating with the protein of interest. Thus, CLIP can only identify sequences with read through of the RT beyond the crosslink site. However, up to 80% of the cDNAs terminate at the crosslinked nucleotide [7–9]. This property has been used to increase the resolution in individual nucleotide resolution crosslinking and immunoprecipitation (iCLIP) [10]. A linker is ligated to the 3′ end of the RNAs, serving as a docking platform for a two-part cleavable RT primer. Circularization of the cDNAs and relinearization places part of the adapter to the 5′ end so that truncated cDNAs are captured for preparation of the RNA-seq libraries.

CLIP techniques have initially been developed for cells cultured in monolayers or mammalian tissue [6, 10]. Thus, it was not clear whether intact plants with UV-absorbing pigments would allow efficient crosslinking, and whether the UV light treatment could elicit UV stress responses in plants that might compromise physiological RNA–protein interactions.

We chose to establish iCLIP for *Arabidopsis*, using *Arabidopsis thaliana* glycine-rich RNA-binding protein 7 (*At*GRP7) as a paradigm. *At*GRP7 is controlled by the circadian clock, an endogenous timekeeper that prepares organisms for the periodic changes of day and night [11]. *At*GRP7 consists of a single RRM and a namesake glycine-rich stretch. The *At*GRP7 transcript oscillates with a peak in the evening, and the oscillations persist in continuous light [12, 13]. Ectopic over-expression of *At*GRP7 (*At*GRP7-ox) leads to damping of the endogenous *AtGRP7* transcript oscillations: Binding of *At*GRP7 to its own pre-mRNA causes a shift to an alternative splice form retaining part of the intron with a premature termination codon (PTC) that is degraded via nonsense-mediated decay (NMD) [14, 15]. Elevated levels of *At*GRP7 also negatively regulate the paralog *At*GRP8 through alternative splicing and NMD. Furthermore, *At*GRP7 regulates alternative splicing of a suite of downstream targets [16]. Additionally, *At*GRP7 functions as an RNA chaperone [17]. Mutation of the conserved Arg49 in the RNA-binding domain (R^49^Q) abolishes in vivo RNA binding and function [18, 19]. *At*GRP7 is involved in a suite of physiological processes, including circadian timekeeping, cold responses, phytohormone responses, and flowering time control [20–22]. To comprehensively understand how *At*GRP7 exerts its diverse functions, determination of its target transcripts and binding landscape at a genome-wide scale is of central importance.

Here, we determined *At*GRP7 targets by iCLIP and a parallel RIP-seq analysis for independent validation. In plants expressing an *At*GRP7-GREEN FLUORESCENT PROTEIN (GFP) fusion we identified significant crosslink sites in 858 target transcripts that were not detected in plants expressing the RNA-binding dead variant *At*GRP7 R^49^Q-GFP, or GFP alone. Of these targets, 452 were also identified by RIP-seq following formaldehyde crosslinking, defining a set of high-confidence binders. In the vicinity of the crosslink sites, UC rich motifs were enriched. To investigate whether the identified in vivo targets are regulated by *At*GRP7 at the mRNA level, we performed total RNA-seq of *At*GRP7 loss-of-function and overexpressing plants. Direct binding targets appear to be predominantly negatively regulated by *At*GRP7. In particular, circadian transcript oscillations are damped in *At*GRP7-overexpressing plants.

## Results

To identify in vivo binding targets of the circadian clock-regulated RBP *At*GRP7 by iCLIP at a genome-wide scale, GFP-tagged *At*GRP7 was expressed under control of its own promoter including the 5′ UTR, 3′ UTR, and intron (*AtGRP7*::*AtGRP7-GFP*) in the *grp7-1* loss-of-function mutant [21]. This construct recapitulates the endogenous expression pattern, thus reducing binding to non-physiological targets due to aberrantly high or ectopic *At*GRP7 expression, and it enables efficient immunoprecipitation using GFP Trap beads with the high affinity single chain antibodies [23].

### Conditions for UV light crosslinking of RNA–protein complexes in *Arabidopsis* plants

To covalently crosslink RNA binding targets to the *At*GRP7-GFP fusion protein in vivo, we adapted UV crosslinking (XL) established for mammalian cells, yeast, or *Caenorhabditis elegans* to *Arabidopsis* plants [10, 24]. Sixteen-day-old *AtGRP7*::*AtGRP7*-*GFP grp7-1* plants were subjected to irradiation with UV-C light (254 nm) at a dose of 500 mJ/cm^2^. To test how this UV-C treatment affects the physiological state of the plants, we first monitored the *METACASPASE 8* (*MC8*) transcript that is known to be upregulated in response to UV stress [25, 26]. We detected a significant increase in *MC8* only after 1 h, indicating that UV stress-induced changes in gene expressions are negligible within the few minutes between treatment and harvest (Additional file 1: Figure S1a, b). As UV-C is also known to trigger the HY5-mediated UV-B signaling pathway, we monitored the level of *HY5 HOMOLOG* (*HYH*), a marker for UV-B photomorphogenesis [27]. *HYH* also showed a significant increase only 60 min after irradiation. The level of the *At*GRP7-GFP fusion protein itself did not change upon UV treatment, excluding false positive results due to a UV effect on *At*GRP7 (Additional file 1: Figure S1c).

As UV-C light also leads to programmed cell death in *Arabidopsis* [28], we checked whether the treatment with 254-nm UV light causes long-term damage by visible inspection of the plants 2, 5, 6, and 8 days after irradiation (Additional file 1: Figure S1d). After 5 days, irradiated leaves showed complete bleaching, but at the same time new, green leaves emerged. Taken together, these data showed that UV light at the dose chosen indeed reaches the interior of the leaves but does not noticeably change the physiological state of the plants in the timeframe before harvest, suggesting that we would obtain a valid snapshot of the *At*GRP7 RNome at the time of irradiation under these conditions.

**Fig. 1:**
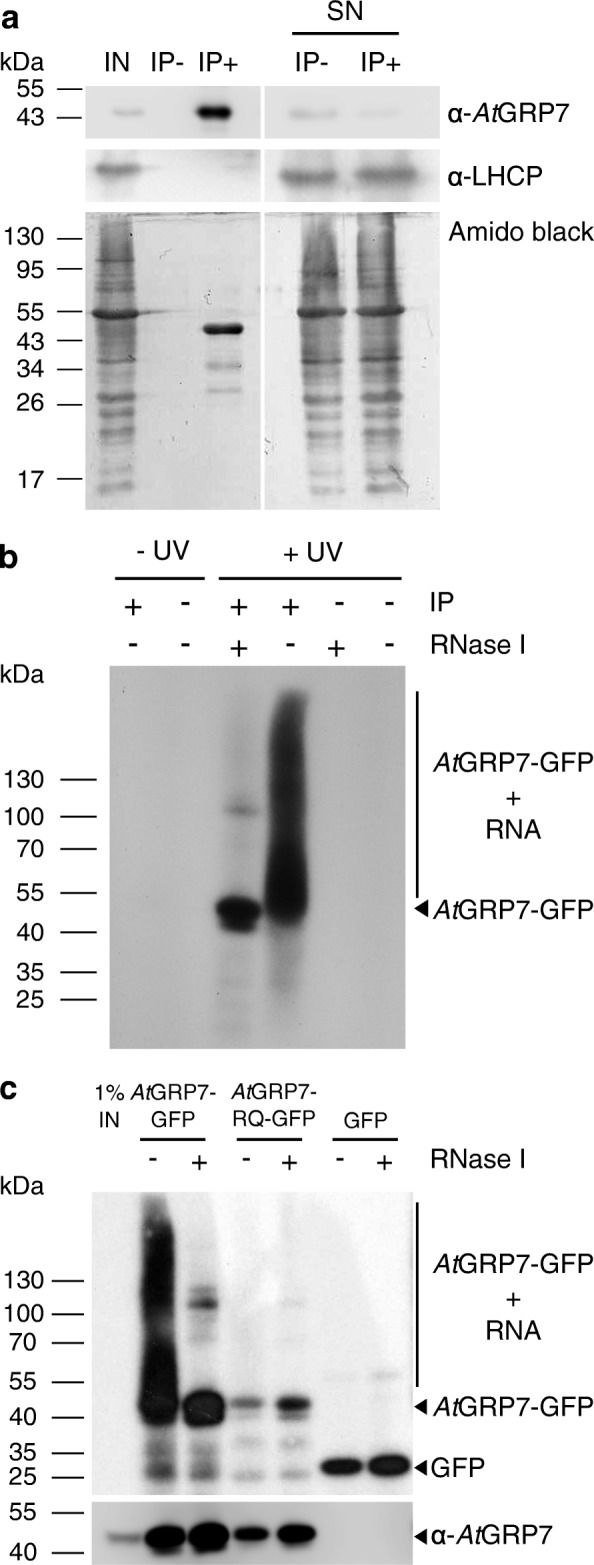
Immunoprecipitation of *At*GRP7 protein–RNA complexes from UV crosslinked *AtGRP7::AtGRP7-GFP grp7-1* plants. RNA–protein interactions were stabilized by UV irradiation of 16-day-old plants with UV light (254 nm) at 500 mJ/cm^2^. Lysates were subjected to immunoprecipitation with GFP Trap beads (*IP+*) and mock precipitation with RFP Trap beads (*IP−*). **a** Aliquots of the lysate (input, *IN*), IP+, IP− and the supernatant (*SN*) of the precipitations were analyzed by immunoblotting with the α-*At*GRP7 antibody. The α-LHCP antibody served as control. For comparison, the membrane was stained with amidoblack. Positions of the molecular weight markers are indicated. **b** Autoradiogram of RNA–protein complexes from *AtGRP7::AtGRP7*-*GFP grp7-1* plants after UV XL and without UV XL and after precipitation (*IP+*) or mock precipitation (*IP−*). Treatment of the precipitate with RNase I (*+ RNase*) indicates the size of the precipitated protein. **c** Autoradiogram of RNA–protein complexes of UV crosslinked *AtGRP7::AtGRP7*-*GFP grp7-1* plants, *AtGRP7::AtGRP7 R* ^*49*^ *Q*-*GFP*, and *AtGRP7::GFP-only* plants. Immunoblot against *At*GRP7 identifies the precipitated protein (*bottom*). Marker positions and the location of the *At*GRP7-GFP RNA adducts are indicated

Next, we confirmed an efficient recovery of the *At*GRP7-GFP fusion protein from the lysate upon precipitation with GFP Trap beads (IP+) but not upon mock precipitation with RED FLUORESCENT PROTEIN (RFP) Trap beads (IP−) by immunoblotting (Fig. 1a). The light harvesting chlorophyll binding protein LHCP was not depleted from the supernatant.

To optimize the yield of the RNA–protein complexes, we adapted the lysate preparation for iCLIP on the basis of the protocol we have developed for RIP following formaldehyde fixation [4, 29]. In particular, higher concentrations of ionic detergent (1% SDS) than used for mammalian cells (0.1% SDS) [10] led to more efficient protein extraction in the lysate. The formation of covalent *At*GRP7-GFP–RNA adducts upon UV crosslinking was monitored by radiolabeling of the RNA. Upon SDS-PAGE, blotting and autoradiography, complexes were detected in crosslinked *AtGRP7*::*AtGRP7*-*GFP grp7-1* plants but not in non-crosslinked plants (Fig. 1b). No complexes were precipitated by RFP Trap beads (IP−) irrespective of UV irradiation. This suggested that the co-precipitating RNAs were mostly targets of the RBP and not only RNAs interacting nonspecifically with the beads. RNase I treatment eliminated most of the crosslinked RNA. As additional controls we used plants expressing the *AtGRP7*::*AtGRP7 R* ^*49*^ *Q*-*GFP* variant with reduced RNA-binding activity due to mutation of a conserved arginine residue in the RRM, or the GFP moiety alone, under control of the *AtGRP7* promoter. Only little RNA–protein complexes were precipitated in these controls (Fig. 1c). Probing of the membrane with the *At*GRP7 antibody confirmed the identity of the precipitated proteins (Fig. 1c).

### iCLIP of *At*GRP7

To comprehensively identify binding substrates of *At*GRP7, libraries were prepared from the RNA–protein complexes for sequencing on the Illumina platform. The procedure is schematically shown in Additional file 1: Figure S2a. *AtGRP7*::*AtGRP7*-*GFP grp7-1* plants were grown in 12 h light–12 h dark cycles, and transferred to continuous light. UV crosslinking was performed after 36 h, at subjective dusk (LL36), the time of *AtGRP7* peak expression. After immunoprecipitation of the RNA–protein complexes from the lysate, the membrane region corresponding to the smear of covalently linked *At*GRP7-GFP–RNA complexes was excised (Additional file 1: Figure S2b). RNA was extracted and libraries were prepared as described in “Methods”. In parallel, negative control libraries were prepared from corresponding regions of lanes containing RNA–protein complexes from GFP-only plants and *AtGRP7*::*AtGRP7 R* ^*49*^ *Q*-*GFP* plants (representative samples shown in Additional file 1: Figure S2b). The read statistics of the individual replicates are shown in Additional file 2: Table S1.

Raw iCLIP reads were subjected to 3′ adapter trimming, quality filtering, and de-multiplexing. PCR duplicates were removed and the barcodes were trimmed. The resulting reads were mapped to the *A. thaliana* TAIR10 reference genome using the additional transcript annotation file atRTD.gff from the reference transcriptome atRTD [30]. Only reads mapping uniquely were kept. Putative crosslink sites were determined separately for each transcript region essentially as described [10]. Only the position one nucleotide upstream of the read start was considered, which represents the XL site. Based on the amount of reads at this site, a false discovery rate (FDR) determined whether an XL site was significantly different from a randomly generated background (see “Methods” for details).

In total, 96,307 significant crosslink sites were identified in the five *AtGRP7*::*AtGRP7*-*GFP grp7-1* replicates. To extract the most robust XL sites, they were required to map to the same position in independent biological replicates. We found 11,021 enriched XL sites in 865 transcripts for *At*GRP7-GFP located at the same position in at least four of the five biological replicates. In the GFP-only plants, 162 significant XL sites were found (0.13% of the XL sites in *AtGRP7*::*AtGRP7*-*GFP grp7-1* plants). These occurred rarely at the same positions in the five independent biological replicates, suggesting that they represent background interactions (Additional file 1: Figure S3). Ten crosslink sites in six transcripts were present at the same position in at least four of the five replicates, and these transcripts were also among the 865 candidate *At*GRP7 targets. In the *AtGRP7*::*AtGRP7 R* ^*49*^ *Q*-*GFP* plants, 349 significant XL sites were identified. Only three XL sites were identified at the same position in two replicates and two of those appeared also in the GFP-only plants (Additional file 2: Table S2). All transcripts with significant XL sites in either GFP-only plants, *At*GRP7 R^49^Q-GFP plants, or both were not considered further, leaving 858 candidate *At*GRP7 target transcripts (Additional file 2: Table S2).

**Fig. 2:**
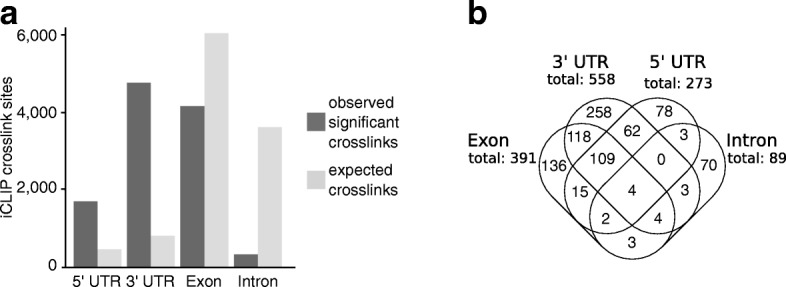
Distribution of the *At*GRP7 crosslink sites within the transcripts. **a** Number of significant crosslink sites (FDR < 0.05) in different transcript regions (*dark grey bars*) compared to a uniform distribution which would be expected according to the cumulative length of the indicated region in the genome based on TAIR10 (*light grey bars*). In all transcript regions a significant difference (*p* < 0.001, hypergeometric) could be observed between the number of observed significant crosslink sites and the number expected for a random distribution according to the size of the region. **b** Venn diagram showing the distribution of the crosslink sites between the different transcript regions. Numbers outside the Venn diagram state the overall number of transcripts with crosslink sites in the respective regions

**Fig. 3:**
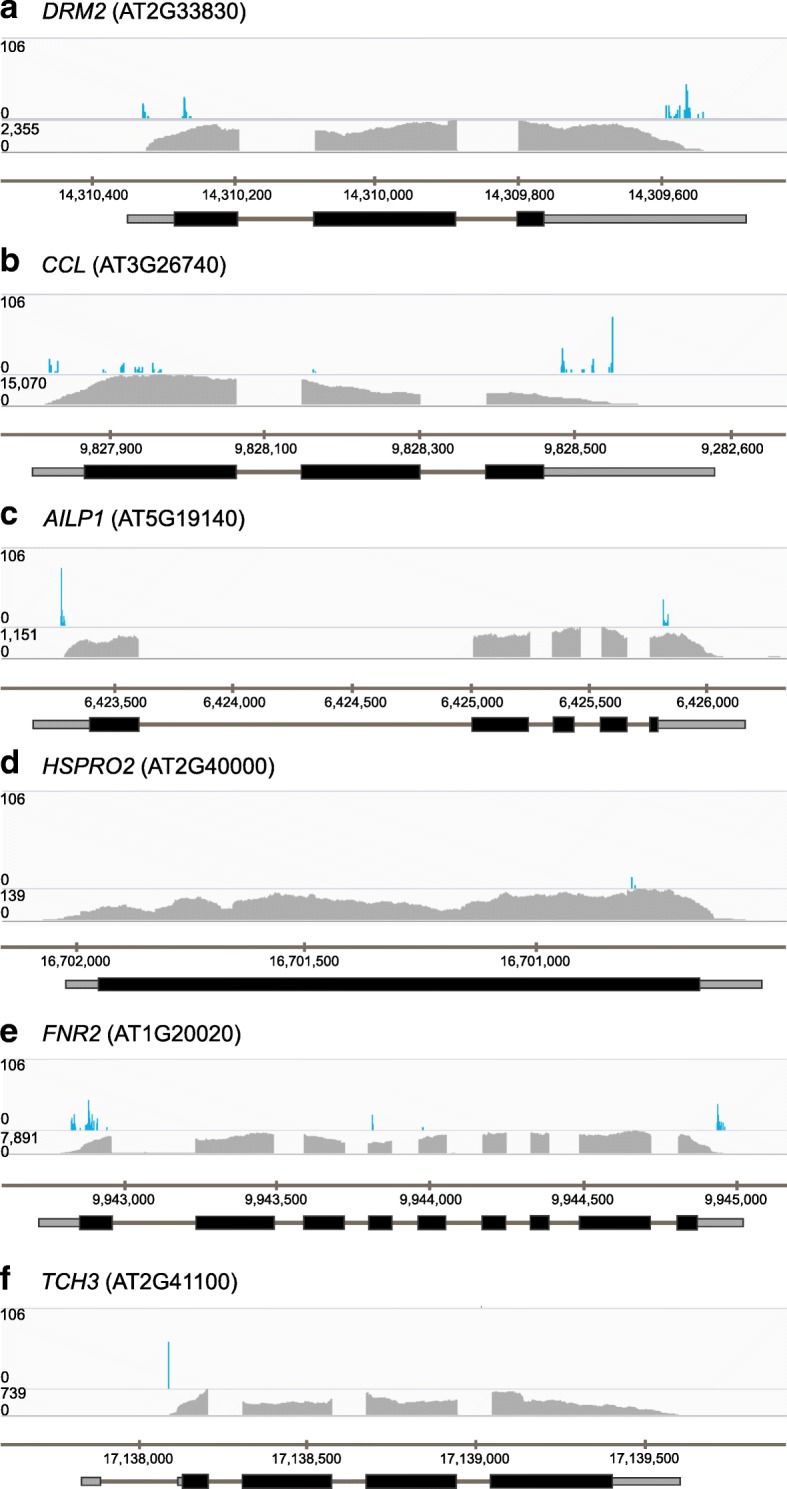
Significant iCLIP crosslink sites on *At*GRP7 target transcripts. Each panel shows the IGV genome browser tracks of significant crosslink sites determined in four out of five biological replicates at LL36 (*top*), the read counts in the LL36 RNA-seq (mean of three biological replicates; *middle*), and the representative gene model and chromosomal position (*bottom*). *Thin bars* represent 5′ UTR (*left*) and 3′ UTR (*right*); *thick bars* denote exons (coding sequences); and *lines* denote introns. The numbers on the x-axis refer to the chromosomal position. **a** *DORMANCY/AUXIN ASSOCIATED FAMILY PROTEIN* (*DRM2*); **b** *CCR-LIKE* (*CCL*); **c** *ALUMINIUM-INDUCED-LIKE PROTEIN 1* (*AILP1*); **d** *ORTHOLOG OF SUGAR BEET HS1 PRO-1 2* (*HSPRO2*); **e** *FERREDOXIN NADP(H) OXIDOREDUCTASE 2* (*FNR2*); **f** *TOUCH3* (*TCH3*)

The localization of the significant XL sites within these transcripts was determined. After normalizing the number of XL sites in each region relative to the overall length of the transcript region in the genome, most XL sites mapped to the 3′ UTR and the 5′ UTR (Fig. 2a). The lowest number was observed in the introns: 258 transcripts contained XL sites only in the 3′ UTR, 136 transcripts only in the exons (coding region), 78 transcripts only in the 5′ UTR, and 70 transcripts only in the introns (Fig. 2b). Many transcripts contained more than one significant XL site, both in the same region and in different regions of the transcripts; e.g., 118 transcripts contained XL sites in both the 3′ UTR and the exons (Fig. 2b). The binding profiles of selected *At*GRP7 targets are shown in Fig. 3 and Additional file 1: Figure S4.

The circadian clock-regulated *DORMANCY/AUXIN ASSOCIATED FAMILY PROTEIN* (*DRM2*; At2g33830) and *CCR-LIKE* (*CCL*; At3g26740) transcripts showed XL sites in the 5′ UTR, exon 1, and the 3′ UTR, and another clock-regulated transcript, *ARABIDOPSIS THALIANA ALUMINIUM-INDUCED-LIKE PROTEIN 1* (*AILP1*; At5g19140), showed XL sites in both the 5′ UTR and 3′ UTR (Fig. 3a–c). *ORTHOLOG OF SUGAR BEET HS1 PRO-1 2* (*ATHSPRO2*; At2g40000), which is involved in basal resistance against *Pseudomonas syringae*, showed XL sites in the exon (Fig. 3d). *FERREDOXIN NAD(P)H OXIDOREDUCTASE 2* (*FNR2*; At1g20020) displayed XL sites in the 5′ UTR, exons 1, 4, and 5, as well as the 3′ UTR (Fig. 3e). For *TOUCH3* (At2g41100), encoding the calcium-binding EF hand family protein TCH3, an XL site was identified in the first intron within the 5´UTR (Fig. 3f).

### Confirmation of high-confidence binders among iCLIP targets by RIP-seq

By adapting iCLIP to *Arabidopsis* plants subjected to UV-C crosslinking, we identified 858 candidate *At*GRP7 target transcripts (Additional file 2: Table S2). However, iCLIP targets are biased towards transcripts with high expression levels, as previously shown for the heterogeneous nuclear ribonucleoprotein hnRNPH1 in HeLa cells [31]. This effect might be attributed to the low crosslinking efficiency upon UV treatment [32]. For example, it has been found that protein–RNA crosslinking occurs on only a minority of contact sites so that crosslinking efficiency can be as low as 1–5% for certain proteins [33]. This contrasts with more efficient formaldehyde crosslinking used in RIP [32]. To compare the spectrum of *At*GRP7 target transcripts identified by these two crosslinking methods and to define a set of high-confidence binders of *At*GRP7, we also performed RIP-seq experiments. *AtGRP7*::*AtGRP7*-*GFP grp7-1* plants grown under the same conditions as for iCLIP were subjected to formaldehyde fixation at LL36. *At*GRP7-GFP and associated RNAs were precipitated with GFP Trap beads and libraries were constructed for sequencing. The read statistics are shown in Additional file 2: Table S3.

To identify transcripts bound to *At*GRP7-GFP (RIP-seq targets) we determined the enrichment of transcripts co-precipitating with *At*GRP7-GFP in the RIP-seq relative to the respective transcript level in poly(A)-containing RNA at LL36, as previously done for the RBPs Wig-1 in human cells and Imp in *Drosophila* [34, 35]. Transcripts with TPM (transcripts per million) < 5 in the RIP-seq libraries were excluded from the analysis, leaving 3602 transcripts. Of those, 2453 showed a log_2_ fold enrichment > 0.5 and q < 0.001 relative to poly(A)-containing RNA, determined by RNA-seq of Col-2 wild type (wt) in three biological replicates, and were therefore considered RIP-seq targets (Additional file 2: Table S4). Using this filtering process, a suite of transcripts were recovered that included the previously identified in vivo targets *AtGRP7*, *AtGRP8*, an Aly/Ref related RNA binding protein/export factor (At5g59950), and a mitochondrial transcription termination factor family protein (At2g36000) [16]. At the same time, transcripts previously shown not to be bound by *At*GRP7 but to be regulated indirectly, such as *AFC2* (encoding a LAMMER-type protein kinase [16]) or the *PATHOGENESIS RELATED1* (*PR1*) transcript [36], were not among the RIP-seq targets, assuring a valid choice of the parameters.

When plotting the average of logarithmic normalized mean counts of each transcript in the RNA-seq data set versus transcripts in the RIP-seq data, we saw an overall positive correlation between RNA-seq counts and RIP-seq counts (Additional file 1: Figure S5a). Nevertheless, many highly expressed genes were not represented in the RIP samples, suggesting that we did not just precipitate highly abundant transcripts nonspecifically. In turn, among transcripts with a high enrichment in RIP-seq were transcripts with a low read coverage in the RNA-seq samples. Thus, binding of *At*GRP7 monitored by RIP-seq does not simply reflect the expression level of the binding substrate. Notably, the number of identified RIP-seq targets for *At*GRP7 is in the same order of magnitude as the 4262 RIP-seq targets identified for the *Arabidopsis* serine/arginine-rich (SR)-like RBP SR45 [5].

To confirm a set of high-confidence binders among iCLIP and RIP-seq targets, we determined the overlap of both data sets (Additional file 2: Table S5); 452 (53%) of the 858 iCLIP targets were also found by RIP-seq, providing an independent confirmation. Conversely, 22.65% of the 2453 RIP-seq targets were also found in iCLIP, whereas 2001 targets were identified only by RIP-seq. Overall, by combining iCLIP with RIP-seq we were able to identify a set of 452 high-confidence binders of *At*GRP7. In parallel, RIP-seq extends the set of *At*GRP7 targets.

### Validation of iCLIP and RIP-seq targets by RIP-qPCR

**Fig. 4:**
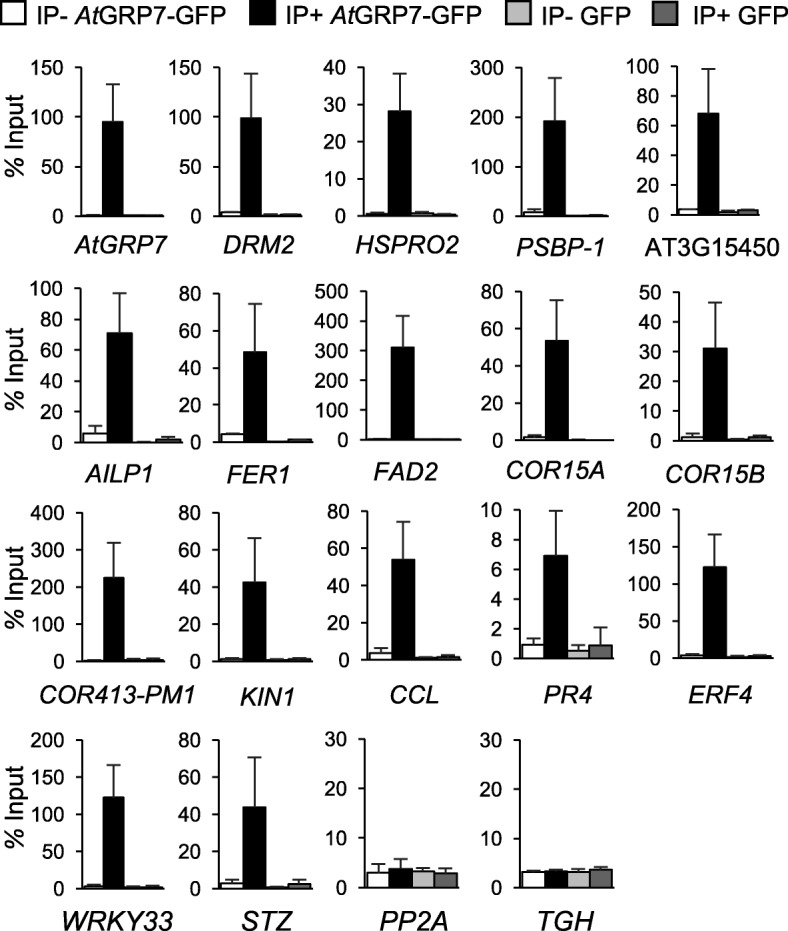
Validation of candidate iCLIP and RIP-seq targets by RIP-qPCR. RIP-qPCR analysis of iCLIP and RIP targets that are circadianly regulated (*AtGRP7*, *DRM2, HSPRO2*, *PSBP-1*, AT3G15450, *AILP1*, *FER1*, and *FAD2*) or cold regulated (*COR15A*, *COR15B*, *COR413-PM1*, and *KIN1*), iCLIP-only targets (*CCL* and *PR4*), and RIP-only targets (*ERF4*, *WRKY33*, and *STZ*) in *AtGRP7::AtGRP7*-*GFP grp7-1* and *AtGRP7::GFP*-only plants. Transcript levels in the GFP Trap precipitate (*IP+*) or RFP Trap precipitate (*IP−*) are presented relative to the transcript levels in the input. Data represent mean ± standard deviation of three biological replicates. *PP2A* and *TGH* as unbound transcripts serve as negative controls

To obtain an independent validation of the targets identified by both iCLIP and RIP-seq, we performed RIP-qPCR on *AtGRP7*::*AtGRP7*-*GFP grp7-1* plants subjected to formaldehyde fixation in three biological replicates. Enrichment upon precipitation with GFP Trap beads but not upon mock precipitation with RFP Trap beads was confirmed for circadian clock-regulated transcripts, *AtGRP7*, *DRM2*, *ATHSPRO2*, the *PSBP-1* transcript encoding the Photosystem II subunit P1 (At1g06680), an aluminum-induced transcript of unknown function (At3g15450), *AILP1*, *FERRETIN1* (*FER1*; At5g01600), *FATTY ACID DESATURASE 2* (*FAD2*), and the cold-regulated transcripts *COR15A* (At2g42540), *COR15B* (At2g42530), *COR413-PM1* (At2g15970), and *KIN1* (At5g15960) (Fig. 4). The transcripts were barely detectable in precipitates from GFP-only plants, confirming low background noise in the control samples. As negative controls we chose *PP2A* and *TOUGH* (At5g23080), encoding an RBP involved in microRNA biogenesis, which were not detected by either iCLIP or RIP-seq. These transcripts were not enriched in RIP-qPCR.

This confirmation of targets suggests that the overlap between iCLIP and RIP-seq represents high-confidence in vivo targets of *At*GRP7. Moreover, binding of transcripts encoding the transcription factors *ETHYLENE RESPONSE FACTOR 4* (*ERF4*; At3g15210), *WRKY33* (At2g38470), and *SALT TOLERANCE ZINC FINGER* (*STZ*; At1g27730), which were identified by RIP-seq only, was validated in independent biological replicates by RIP-qPCR. Their absence from the iCLIP targets could be due to the reduced crosslinking efficiency of UV light or because they represent indirect targets.

The circadian clock-regulated *CCR-LIKE* (*CCL)* transcript and *PATHOGENESIS RELATED 4* (*PR4*), encoding a protein with similarity to the antifungal chitin-binding protein hevein from rubber tree latex, had been identified only by iCLIP (Additional file 2: Table S2). They were significantly enriched in RIP-qPCR with GFP Trap beads but not with RFP Trap beads. The confirmation by RIP-qPCR indicates that these transcripts can indeed be crosslinked to *At*GRP7 by formaldehyde.

For some of the transcripts, the level of precipitated RNA seems higher than expected based on the input (Fig. 4). This observation is not unprecedented and may be due to different efficiencies in RNA extraction in immunoprecipitated fractions compared to total extract as well as a higher efficiency of reverse transcriptase on lower amounts of RNA [3, 37].

### Determination of the *At*GRP7 binding landscape

A key advantage of iCLIP is its precise mapping of the interaction site, thereby allowing the prediction of a binding motif. Consequently, the next step was the investigation of candidate *At*GRP7 binding motifs in the vicinity of the crosslink sites. The presence of XL sites in 5′ UTRs, exons, introns, and 3′ UTRs could imply different functions of the protein on these regions. Thus, motifs were generated for each transcript region separately.

**Fig. 5:**
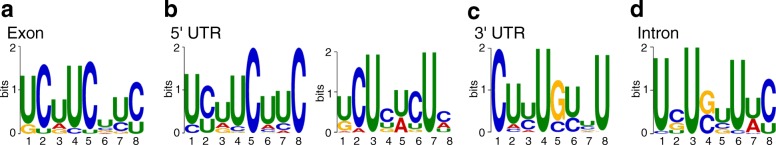
Most significant binding motifs identified by MEME analysis. The most significant motifs (based on their *p* value) identified by MEME analysis of the 21-nucleotide region surrounding the significant crosslink sites (FDR < 0.05) occurring in at least four out of five replicates in **a** exons, **b** 5′ UTRs, **c** 3′ UTRs, and **d** introns of binding targets at LL36

The genomic sequence at the XL sites was extended by ten nucleotides in either direction and used to identify conserved motifs with the MEME-Suite [38]. To discriminate against random binding events, a background was generated by simulating the identical number of crosslinks uniformly in the corresponding 5′ UTR, exon, introns, and 3′ UTR, respectively. The simulated XL sites were extended and genomic sequences extracted, exactly as for the observed XL sites. The resulting significant motifs were generally U/C-rich. One significant motif was obtained in the exons, introns, and 3′ UTR, respectively, and two for the 5′ UTR (Fig. 5). To perceive differences between the motifs identified by MEME, we performed a clustering analysis based on pairwise comparison between motifs across regions using the R package *DiffLogo* (Additional file 1: Figure S6a). In the resulting distance tree, the exon and 5′ UTR motifs clustered together and consequently were closely related. In contrast, the 3′ UTR motif is located outside this subgroup and therefore exhibited the largest difference from the other motifs. This may indicate a variation in binding preference for *At*GRP7 between the transcript regions and point to different functions of the protein depending on the region.

In a complementary approach, we determined enriched pentamers in the vicinity of the XL sites essentially as previously described [10]. Again, the XL site was extended by ten nucleotides in either direction. A five-nucleotide-frame was shifted along these sequences, all pentamers within these frames were recorded, and enriched pentamers were calculated with a Z-score analysis. For the 5′ UTR and the exon, the evaluation of pentamer frequencies around the XL sites also yielded U/C-rich sequences (Additional file 2: Table S6). Similar to the MEME analysis, the pentamers in the 3′ UTRs were U-rich as well, but the pentamer analysis revealed a higher frequency of G than the MEME motif. For introns, the pentamer frequencies differed slightly from the MEME results, being more C/G-rich.

So far, in vitro binding requirements of recombinantly expressed *At*GRP7 have been investigated for the 3′ UTR and intron of its own transcript, using electrophoretic mobility shift assays and fluorescence correlation spectroscopy [14, 18, 39]. For the 3′ UTR, iCLIP now identified three significant crosslink sites around a 32-nucleotide sequence previously used for binding studies (Additional file 1: Figure S7a). Notably, upon scanning the *AtGRP7* sequence for the significant 3′ UTR motif using FIMO [38], several matches were found within or close to the binding region defined in vitro. Furthermore, sequences corresponding to the pentamers enriched in the 3′ UTR were found in this 32-nucleotide region, including one that overlapped a minimal *At*GRP7 binding sequence delineated by a deletion analysis [39]. Thus, a known in vitro binding site was confirmed in vivo.

Furthermore, the iCLIP data revealed *At*GRP7 binding to the first half of the intron located upstream of the cryptic 5′ splice site. Several matches of the MEME intron motif were observed near the XL sites (Additional file 1: Figure S7b). An additional *At*GRP7 binding site had been determined within the second half of the intron in vitro [14, 40]. No significant XL sites were obtained in this region. However, an intron motif was detected next to the determined in vitro binding site [40]. This binding site may not be accessible for *At*GRP7 in vivo under the conditions analyzed.

### Impact of *At*GRP7 on candidate targets

Identification of in vivo targets represents a first step towards understanding posttranscriptional networks controlled by RBPs. The consequences the binding may have for the transcriptome are usually assessed by knocking down the RBP and monitoring the fate of the target mRNAs [41–43]. To unravel whether the in vivo targets we have identified are regulated by *At*GRP7 at the RNA level, RNA-seq was performed on a loss-of-function mutant. Because the *grp7-1* T-DNA line has elevated *AtGRP8* levels due to relief of repression by *At*GRP7, we used the *grp7-1 8i* line that has an RNAi construct against *At*GRP8 and expresses *AtGRP8* at levels comparable to wt plants [21]. As the redundancy between *At*GRP7 and *At*GRP8 may mask a clear loss-of-function phenotype, we included plants with constitutively elevated *At*GRP7 levels (*At*GRP7-ox) in the analysis. Libraries were prepared from plants harvested in parallel to the samples used for RIP-seq at LL36. The read statistics are presented in Additional file 2: Table S7. Only transcripts with a TPM > 1 in at least one of the genotypes (average of three biological replicates) were considered. Transcripts with q < 0.05 were considered significantly differentially expressed either between mutant and wt, or between *At*GRP7-ox plants and wt (differentially expressed genes (DEGs)). The list of DEGs was cross-referenced against the iCLIP and RIP-seq targets (Additional file 1: Figure S8a, b; Additional file 2: Table S8). Of the iCLIP targets, 93 were significantly differentially expressed in *grp7-1 8i* (of 2087 DEGs) and 534 in *At*GRP7-ox plants (of 7855 DEGs); 62 transcripts were DEGs in both genotypes. When considering the high-confidence binders that were also identified by RIP-seq, 58 were significantly differentially expressed in *grp7-1 8i*, 293 in *At*GRP7-ox, and 38 in both genotypes. For targets identified by RIP-seq, 365 were DEGs in *grp7-1 8i*, 1207 in *At*GRP7-ox, and 157 in both genotypes. The considerably lower number of DEGs in the loss-of-function mutant than in the *At*GRP7-ox plants is at least partly due to the redundancy of *At*GRP7 and *At*GRP8, as seen before [16, 44].

**Fig. 6:**
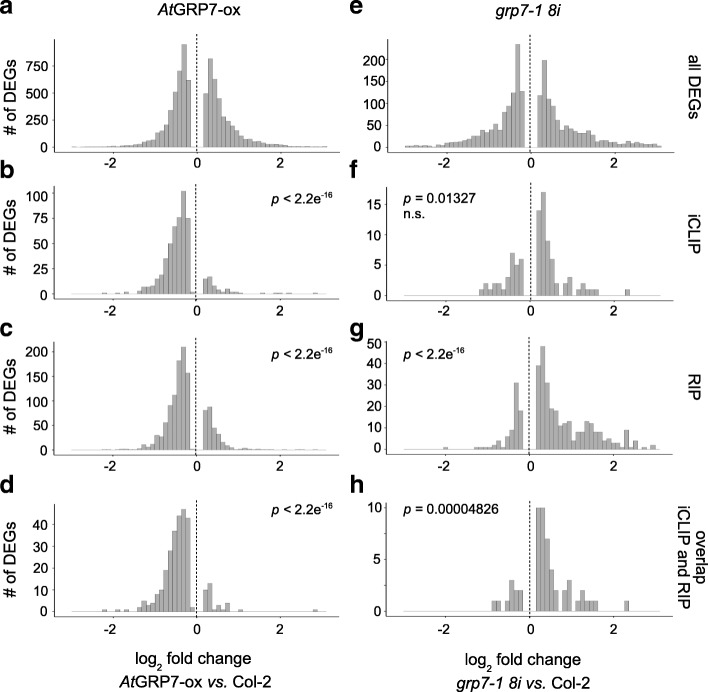
Changes in distribution of the log_2_ fold changes of genes differentially expressed at LL36 (DEGs) in the *grp7-1 8i* mutant or *At*GRP7-ox plants upon binding to *At*GRP7. Log2 fold change distribution of all genes differentially expressed at LL36 (DEGs) from the RNA-seq data set (**a** *At*GRP7-ox, **e** *grp7-1 8i*), as well as of iCLIP targets with a significant differential expression (**b** *At*GRP7-ox, **f** *grp7-1 8i*), RIP targets with a significant differential expression (**c** *At*GRP7-ox, **g** *grp7-1 8i*), and high-confidence binders identified by both iCLIP and RIP (**d** *At*GRP7-ox, **h** *grp7-1 8i*). The distribution of all identified DEGs in RNA-seq (**a**, **e**) was tested pairwise against all target groups. The resulting *p* value is displayed accordingly

Overall, a similar proportion of transcripts were either upregulated or downregulated in *At*GRP7-ox plants or the *grp7-1 8i* mutant, respectively (Fig. 6a, e). Notably, significantly more of the differentially expressed iCLIP targets were downregulated in the *At*GRP7-ox plants than upregulated (Fig. 6b). This was also seen for the RIP-seq-only targets or the high-confidence binders identified by both iCLIP and RIP-seq (Fig. 6c, d). In contrast, the differentially expressed iCLIP targets, RIP-seq targets, and high-confidence binders were preferentially upregulated in the *grp7-1 8i* line (Fig. 6f–h). This indicates that DEGs bound in vivo by *At*GRP7 are mostly under negative control by *At*GRP7. In contrast, some of the genes that are positively regulated by *At*GRP7 may be controlled indirectly via intermediate factors. For example, the defense-related *PR1* transcript is upregulated by elevated *At*GRP7 levels through NPR1-dependent activation of the promoter, and accordingly, the *PR1* transcript is not an in vivo target [36].

The differential expression of high-confidence binders was validated by RT-qPCR in independent samples (Additional file 1: Figure S8c). Considering the involvement of *At*GRP7 in the response to diverse stress factors, we selected candidate targets associated with stress responses. The cold-responsive *COR15A* transcript encoding a chloroplast protein of unknown function was reduced in *At*GRP7-ox plants. *FAD2* encoding an ER-localized ω6 desaturase required for salt tolerance was also reduced [45]. The level of the clock-regulated *DRM2* was reduced, as was *FER1. ATHSPRO2* was elevated in *grp7-1 8i* compared to wt. Furthermore, a suite of transcription factors of the AP2/ETHYLENE RESPONSE FACTOR and WRKY families, which were only identified by RIP-seq, were expressed at higher levels in *grp7-1 8i* compared to wt (Additional file 1: Figure S8d). Among those were *ERF4*, which is associated with jasmonic acid, ethylene, and abscisic acid signaling, and the zinc finger factor S*TZ*, which plays both a positive and negative role in the tolerance of plants to salinity, heat, and osmotic stress [46]. Furthermore, *WRKY33*, which regulates the antagonistic relationship between defense pathways mediating responses to *Pseudomonas syringae* and necrotrophic fungal pathogens, respectively, is elevated in *grp7-1 8i*. It remains to be determined whether in vivo binding of *At*GRP7 to *HSPRO2* or *WRKY33* and the differential expression of these genes relates to the role of *At*GRP7 in plant innate immunity [19, 36, 47, 48]. Overall, the differential expression of the selected DEGs measured by RT-qPCR correlated well with the log_2_ fold change in the RNA-seq data (Additional file 1: Figure S8c, d).

### *At*GRP7 regulates circadian target transcripts

Among the *At*GRP7 iCLIP and RIP targets was the circadian clock regulated *AILP1*, previously identified by fluorescent differential display as aberrantly expressed upon *At*GRP7 overexpression [18]. To determine the impact of *At*GRP7 on clock-regulated transcripts globally, the targets were compared to a list compiling 5230 circadianly regulated *Arabidopsis* transcripts, presented in [49]. Indeed, 205 of the 452 high-confidence binders (45%), 383 of the 858 iCLIP targets (45%), and 924 of the 2453 RIP targets at LL36 (38%) were circadianly regulated according to [49]. In contrast, of all the genes expressed at LL36 in our RNA-seq data, only 23% were circadianly regulated according to [49].

**Fig. 7:**
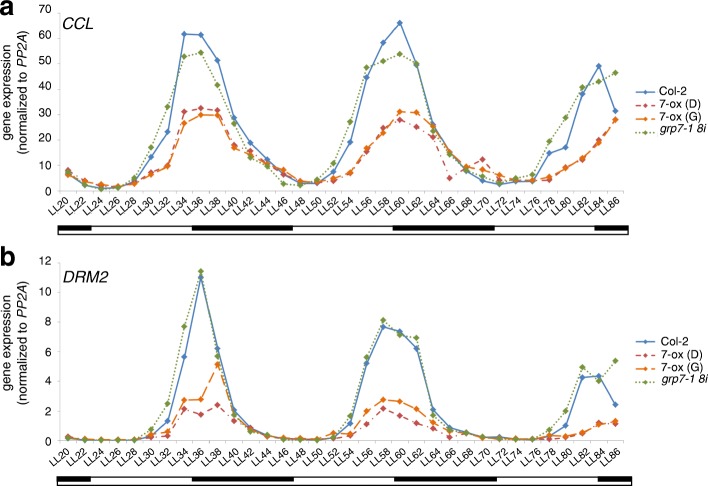
Circadian expression of *CCL* and *DRM2* measured in 2-h time intervals. Seedlings were grown in 12-h light and 12-h dark phase for 16 days and then transferred to constant light (*LL*). Transcript levels of **a** *CCL* (AT3G26740) and **b** *DRM2* (AT2G33830) were determined every 2 h in wt (Col-2), two independent *At*GRP7 overexpressing lines (7-ox), and the *grp7-1 8i* mutant. The time course was performed twice with similar results. *Open bar*, constant light; *inserted dark bar*, subjective night

Therefore, we monitored the circadian expression patterns of iCLIP targets differentially expressed in the *At*GRP7-ox plants with high temporal resolution. Plants grown in 12 h light–12 h dark cycles were transferred to constant light and harvested at 2-h intervals, starting at LL20. *CCL* and *DRM2* showed a reduced peak in two independent transgenic *At*GRP7-ox lines on three consecutive days, indicating that *At*GRP7 indeed negatively regulates the oscillations of these transcripts. Although one would expect an opposite phenotype in the *grp7-1 8i* line, the oscillations were similar to wt plants (Fig. 7), suggesting *At*GRP7 acts redundantly with other factors in shaping these transcript oscillations.

### *At*GRP7 targets in the subjective morning

To test whether the spectrum of *At*GRP7 targets depends on the time of the day, we performed another iCLIP experiment on *AtGRP7*::*AtGRP7*-*GFP grp7-1* plants harvested 12 h out of phase, at LL24. The read statistics are presented in Additional file 2: Table S9. After processing the reads as done for the LL36 iCLIP targets, we arrived at 469 transcripts with significant XL sites in at least two of the three *AtGRP7*::*AtGRP7*-*GFP grp7-1* replicates and absent in the GFP-only plants and *AtGRP7*::*AtGRP7 R* ^*49*^ *Q*-*GFP* plants (Additional file 2: Table S10). A similar distribution of XL sites in the different regions of the transcript was found as for the transcripts bound in the subjective evening, at LL36 (Additional file 1: Figure S9b). After normalizing to the length of the transcript region, the highest number of XL sites again was found in the 3′ UTRs (Additional file 1: Figure S9a).

Of the transcripts identified in iCLIP, 386 were bound at both time points. The distribution of XL sites was similar at LL24 and LL36, e.g., for *KIN1*, *COR27*, *COR413-PM1*, or *FNR2* (cf. Additional file 1: Figure S4). Other examples were *NITRATE REDUCTASE 2* (*NIA2*; At1g37130), where LL24 and LL36 share a similar XL site in the 3′ UTR, and *POLY A BINDING PROTEIN 2* (*PABP2*; At4g34110), which contains a group of XL sites in the 5′ UTR (Additional file 1: Figure S9c, d).

As done for LL36, enriched motifs in the vicinity of XL sites were determined by MEME in the LL24 targets (Additional file 1: Figure S10). As seen before, the significant motifs for the exon and the 5′ UTR are U/C rich. When clustering the motifs of both time points with *DiffLogo* [50], the LL24 and LL36 motifs of these regions were grouped together (Additional file 1: Figure S6b). Similarly, the motifs for the intron at both time points were more closely related to each other than to the other motifs of the same time point. The same effect was observed for the 3′ UTR, indicating that the bound motifs in the different transcript regions were highly similar for the subjective morning and the subjective evening.

Independent validation of the iCLIP targets was performed by RIP-seq of *AtGRP7*::*AtGRP7*-*GFP grp7-1* plants subjected to formaldehyde fixation at LL24. The read statistics are displayed in Additional file 2: Table S11. Of the 2256 RIP-seq targets with a log_2_ fold enrichment > 0.5 and q < 0.001 over poly(A) RNA (Additional file 2: Table S12), 196 targets were identified also by iCLIP (Additional file 2: Table S13). Thus, 42% of the 469 iCLIP targets were also identified by RIP and, conversely, 8.7% of the 2256 RIP-seq targets were also identified by iCLIP.

To unravel an impact of *At*GRP7 on the LL24 targets, we cross-referenced the targets against RNA-seq data generated in *grp7-1 8i* and *At*GRP7-ox plants harvested at LL24. The read statistics are shown in Additional file 2: Table S14. Again, transcripts with q < 0.05 were considered significant DEGs between either mutant and wt or *At*GRP7-ox plants and wt (Additional file 2: Table S15; Additional file 1: Figure S11). Of the iCLIP targets, 24 were significantly differentially expressed in *grp7-1 8i* (of 731 total DEGs in the mutant), and 306 in *At*GRP7-ox plants (of 5927). Eighteen transcripts were DEGs in both genotypes. When considering the high-confidence binders that were also identified by RIP-seq, 14 were DEGs in *grp7-1 8i*, 147 in *At*GRP7-ox plants, and nine in both. For targets identified by RIP-seq, 177 were DEGs in *grp7-1 8i*, 996 in *At*GRP7-ox, and 51 in both. As observed for LL36, DEGs bound in vivo by *At*GRP7 are mostly under negative control by *At*GRP7 at LL24 (Additional file 1: Figure S12).

Similar to LL36, the binding targets at LL24 were enriched for circadianly regulated transcripts, as listed in [49]: 97 of the 196 high confidence binders (49%), 231 of the 469 iCLIP targets (49%), and 876 of the 2256 RIP targets (39%) at LL24 are circadianly regulated. In contrast, only 23% of all transcripts expressed at LL24 in our RNA-seq were circadianly regulated.

### Alternative splicing targets identified by iCLIP and/or RIP-seq

Previously, we identified 59 alternative splicing events significantly changed in response to elevated *At*GRP7 levels [16]. To monitor splicing changes globally, we analyzed the RNA-seq data of the *grp7-1 8i* mutant and *At*GRP7-ox plants using *SUPPA* [51]. *SUPPA* determines the percent spliced in (PSI) value, defined as the ratio between the TPM of the isoform including the event and the overall TPM of all isoforms for single splicing events. Changes of more than 10% in the isoform ratio (|ΔPSI| > 0.1) between the *grp7-1 8i* mutant and wt or *At*GRP7-ox and wt, respectively, with *p* < 0.01 were considered significant (Additional file 2: Table S16). Selected events were validated by RT-PCR.

The most common type of alternative splicing events affected in both genotypes was intron retention. The transcript encoding the calcium-binding EF hand family protein TCH3 was found in both RIP and iCLIP at LL36. In the *grp7-1 8i* mutant, more intron retention in the first exon was detected (Additional file 1: Figure S13a). The location of 5' UTR XL sites close to the intron retention event suggests an effect of *At*GRP7 binding on the splicing event. For the iCLIP target *FNR2* the first intron, which contains a PTC, was retained more often in *At*GRP7-ox plants than in the wt and the XL site mapped in close proximity of the event (Additional file 1: Figure S13b). RIP-RT-PCR using primers that detect both isoforms showed that *At*GRP7 preferentially binds to the spliced isoform (Additional file 1: Figure S14). The At1g28580 transcript encoding a GDSL-like lipase was identified by RIP-seq at LL36. Elevated *At*GRP7 levels promote intron retention in the first exon, also leading to a PTC. The increase in the intron retained band was confirmed (Additional file 1: Figure S13c). At5g66240 encodes a Transducin/WD40 repeat-like superfamily protein and was identified by RIP-seq at LL36. An increase in *At*GRP7 levels leads to both an increased steady-state abundance and less retention of intron 1, predicted to remove three amino acids (Additional file 1: Figure S13d). RIP-RT-PCR showed that *At*GRP7 binds to both isoforms (Additional file 1: Figure S14). At3g17100 encodes the transcription factor ATBS1 INTERACTING 3 (AIF3), detected by RIP-seq at both time points. Increased *At*GRP7 levels led to retention of intron 1 in the 5′ UTR (Additional file 1: Figure S13E). RIP-RT-PCR showed that *At*GRP7 binds to the small isoform (Additional file 1: Figure S14).

Another splicing event, exon skipping, was also found among the iCLIP and RIP-seq targets. An example for a RIP-seq target is *FAX4* (At1g33265), which encodes a fatty acid exporter in the chloroplast membrane. Upon *At*GRP7 overexpression, exon 2 skipping was promoted (Additional file 1: Figure S13f), leading to a frameshift and a PTC, which causes the predicted protein to lose a polyleucine stretch and three transmembrane helices. RIP-RT-PCRs confirmed that *At*GRP7 interacts at least with the longer isoform, which retains the alternative exon (Additional file 1: Figure S14).

Furthermore, we found alternative usage of 5′ or 3′ splice sites among the *At*GRP7 targets. The RIP-seq target At1g76020 encodes a thioredoxin superfamily protein, where an elevated *At*GRP7 dosage leads to a shift to an alternative 5′ splice site and the retention of 88 nucleotides of the first intron (Additional file 1: Figure S13g). Again, the event introduces a PTC and the isoform is a predicted NMD target [52]. RIP-RT-PCR showed that *At*GRP7 binds to the fully spliced isoform (Additional file 1: Figure S14). For all these splicing events the isoform ratio in wt was similar at LL24 and LL36, indicating that these events are not under circadian control (Additional file 2: Table S17). At LL24, mis-expression of *At*GRP7 had a similar impact on these splicing events as observed at LL36 (Additional file 1: Figure S15; Additional file 2: Table S17). Only for *TCH3* was the increased intron retention in the mutant not statistically significant at LL24 in contrast to LL36. This indicates that the effect of *At*GRP7 on these investigated alternative splicing events in bound targets was not time-of-day dependent.

Several transcripts with splicing events previously shown to be regulated by *At*GRP7 were also identified here [16]. In addition to *AtGRP7* and *AtGRP8*, At2g36000, encoding a mitochondrial termination factor family protein with an intron in its 3′ UTR that is spliced in a temperature-dependent manner [53], the Aly/Ref related RNA binding protein/export factor (At5g59950), the chaperone DnaJ (At3g62190), the ankyrin repeat-containing protein AKR2 (At4g35450), and the cofactor for nitrate reductase CNX7 (At4g10100) were found by RIP-seq.

As *SUPPA* does not reveal the differential usage of polyadenylation sites, we analyzed selected examples separately with RT-PCR. At1g45474, encoding the LHCA5 protein of the light harvesting complex of photosystem 1, was among the RIP-seq targets and combines an alternative 5′ splice site with skipping of the last exon, leading to an alternative 3′ UTR. Both, the wt and *grp7-1 8i* showed a preference for the distal polyadenylation site, whereas in *At*GRP7-ox plants the distal and proximal polyadenylation sites were used similarly (Additional file 1: Figure S13h). The thiamine biosynthetic enzyme *THIAMIN C* (*THIC*; At2g29630), an iCLIP and RIP-seq target, is known to undergo alternative polyadenylation as well. Similar to *LHCA5*, the event combines an alternative 5′ splice site with skipping of the last exon. Additionally, *THIC* harbors a Thiamin-dependent riboswitch in the 3′ UTR [54]. An increasing *At*GRP7 level influences alternative splicing of intron 6, which leads to a decrease of the ID9 isoform polyadenylated at the distal site (Additional file 1: Figure S13i). This isoform contains the majority of the riboswitch, without the initial eight nucleotides, and is known to be less stable. In contrast, isoform 1 is more stable, lacks the riboswitch, and leads to proximal polyadenylation. *At*GRP7 binds to both isoforms (Additional file 1: Figure S14). Overall, these show that *At*GRP7 impacts different types of splicing events by direct in vivo binding to the targets and that it can affect alternative polyadenylation through its impact on splicing of the penultimate exon.

## Discussion

Here we present the first iCLIP analysis identifying in vivo targets of plant RBPs and their binding landscape at a genome-wide scale. For the circadian clock regulated RBP *At*GRP7 we identify 858 transcripts with significant crosslink sites present at the same position in at least four out of five biological replicates of UV crosslinked *AtGRP7::AtGRP7-GFP grp7-1* plants, and absent in plants expressing GFP-only or an RNA-binding-dead variant of *At*GRP7. Of these iCLIP targets, 53% were also independently identified by RIP-seq, and thus represent a set of high-confidence binders.

### Detection of iCLIP targets and discrimination against background

iCLIP has become a state-of-the-art method to study RNA–protein interactions in vivo; to date, however, the successful application of iCLIP in *Arabidopsis* has not been reported. iCLIP critically relies on UV-C crosslinking. Thus, we first tested this step and its effect on *Arabidopsis* seedlings, as it was not clear whether only transcripts present in the top cell layers of the leaf would be crosslinked. The UV dose of 500 mJ/cm^2^ used in our experiments is in the same range as the 100 or 150 mJ/cm^2^ used in mammalian cells [10, 55, 56], 250 mJ/cm^2^ used in *Drosophila* [35], and 400 mJ/cm^2^ used in neuronal cells [43].

We found that irradiated leaves eventually bleached, indicating that the UV light indeed reaches the interior of the leaves. The emergence of new leaves several days after irradiation may indicate that the UV light does not reach deeply into the meristem and thus transcripts expressed there may not be crosslinked efficiently. Additionally, we compared the iCLIP targets with transcripts that have been shown to be preferentially expressed in the mesophyll or the vasculature of 9-day-old seedlings based on a more than tenfold enrichment in these tissues compared to the average transcript level in total leaves [57]. Of 250 transcripts preferentially expressed in the mesophyll, 51 were recovered by *At*GRP7 iCLIP at LL36, with 21 of those also found by RIP-seq. Of the 280 transcripts preferentially expressed in the vasculature, three were found by iCLIP and two also by RIP-seq. Among the iCLIP targets we also find plastidic carboanhydrase *CA1* (At3g01500), established as a marker for mesophyll cells [58]. The identification of transcripts in these tissue layers indicates that the UV treatment crosslinks transcripts in the interior of the leaf.

On the other hand, transcript levels of UV stress response marker were not elevated in the timeframe relevant for the experimental procedure and thus the physiological state of the plants should not be grossly altered. It cannot be ruled out that posttranslational modifications, e.g., in response to UV-activated kinase signaling, impacts the RNA binding properties of RBPs, a limitation inherent in all CLIP studies. However, UV crosslinking occurs on a very fast time scale, and any modification occurring after the formation of the covalent bonds does not influence the spectrum of targets. Recently, irradiation with 254 nm UV light has also proven successful for crosslinking mRNAs and bound proteins in studies aimed at identifying all mRNA interacting proteins in *Arabidopsis* cell cultures, protoplasts, leaves, or etiolated seedlings [59–61].

We used the strategy developed by König et al. [10] to determine the XL sites, where significant XL sites were discriminated against a randomly generated background using FDR. To select the most reliable targets, we applied a stringent filtering criterion, requiring that the significant XL sites were called in at least four out of five independent biological replicates. When we increased the stringency and considered XL sites present at the same position in all five replicates, some of the known and confirmed *At*GRP7 targets were not present any more. As RNA-binding domains contact more than one nucleotide in the RNA, it seems plausible that the XL sites of the different replicates disperse and do not necessarily map to the identical nucleotide. Requiring the XL sites of all five replicates to map to the exact same position eliminates those sites that might scatter around a few nucleotides.

Furthermore, we excluded transcripts from the analysis that contained significant XL sites in plants expressing either GFP alone or *At*GRP7 R^49^Q-GFP under control of the *AtGRP7* promoter. Overall, there was a much reduced number of XL sites in the controls compared to the *At*GRP7-GFP plants, and the XL sites did not map frequently to the very same positions in the independent replicates (Additional file 1: Figure S3). The GFP protein has recently also been found to be a suitable negative control in mammalian iCLIP studies [62, 63]. Among the transcripts appearing in the controls were several tRNAs. Furthermore, transcripts encoding the small subunit of RIBULOSE BISPHOSPHATE CARBOXYLASE, one of the most abundant plant proteins, appeared in the controls at time point LL24. As more studies become available for *Arabidopsis* RBPs, the experience with frequently observed background XL sites will increase [64].

### iCLIP and RIP-seq identify an overlapping but not identical set of target transcripts

Global RIP-seq confirmed 53% of the iCLIP targets at LL36 and 43% at LL24, yielding a set of high-confidence binders. This is in the same range as observed for mammalian hnRNPH1 in a comparison of iCLIP and RIP which was performed without formaldehyde crosslinking [31]. RIP found 32% of iCLIP targets of hnRNPH1, and in turn, 28% of the hnRNPH1 RIP targets were identified by iCLIP [31]. The identification of several hundred iCLIP targets indicates that *At*GRP7 binds to a wide range of transcripts. Accordingly, *At*GRP7 is one of the few proteins discovered in all three studies identifying mRNA interacting proteins globally in cell cultures, protoplasts, leaves, or etiolated seedlings [65].

Besides the high-confidence binders identified by iCLIP and confirmed by RIP-seq, we identified a suite of transcripts either by iCLIP only or RIP-seq only and validated a selection by RIP-qPCR. The identification of overlapping but not identical sets of targets indicates that both methods are complementary and generally enhance the identification of RBP targets. Crosslinking by formaldehyde and by UV light both have distinct advantages and drawbacks. UV does not crosslink proteins, thus limiting the analysis to RNAs immediately bound by the RBPs, whereas formaldehyde fixation yields direct and indirect targets of RBPs. In turn, formaldehyde has a higher crosslinking efficiency and thus may identify binding targets of lower abundance [32].

To identify RIP-seq targets we filtered for transcripts enriched in the precipitate over poly(A) RNA rather than enrichment relative to transcripts co-precipitating with GFP only or in a mock precipitation with RFP Trap beads. Our rationale was that the amount of RNAs co-precipitating with GFP alone was below the detection limit for photometric and fluorometric quantification. Therefore, we expected that precipitation of GFP-only plants or mock precipitation of *At*GRP7-GFP with RFP Trap beads would produce low complexity libraries and consequently have a low coverage [66]. This is in line with our previous RIP-qPCR experiments and our validated data, showing low background of unspecific binding to mock controls (IP−) or GFP only. Similar results were obtained after mock precipitation with RFP Trap beads (IP−).

So far, two genome-wide investigations on RBP targets have been reported in *Arabidopsis*. RIP-seq on formaldehyde-treated plants expressing the serine/arginine-rich (SR)-like RBP SR45 fused to GFP identified 4262 SR45-assosciated RNAs, designated SARs, that were enriched upon precipitation with GFP antibodies from the SR-GFP plants over wild-type plants based on three biological replicates [5]. Of the SAR genes, 116 were differentially expressed in the *sr45-1* mutant relative to the SR45.1-GFP line. A CLIP study of HLP1, an hnRNP A/B-like protein, identified 9031 binding sites from the sense transcripts of 5569 genes in one biological replicate [67]. Binding sites showed overrepresented A-rich and U-rich motifs predominantly near the poly(A) sites. Mutation of HLP1 causes altered polyadenylation in 429 of the 5569 target transcripts, including the flowering time gene *FCA*. Of interest, HLP1 binds also to the *AtGRP7* 5′ UTR, although the consequences are not yet known [67].

### Binding mode of *At*GRP7

Because the iCLIP reads end at the XL site, insights can be obtained into motifs recognized by the RBPs with high resolution. *At*GRP7 binds to all transcript regions with a preference for the 3′ UTR. The lowest number of XL sites mapped to the intron. This may also relate to the fact that introns are less represented in whole cell lysates as used for iCLIP here. The presence of significant XL sites in different regions of the transcripts points to different functions *At*GRP7 may fulfill when binding to different regions. The U/C-rich motifs identified in the 5′ UTR and exon by MEME were closely related, whereas they shared low similarities with the motif identified in the 3′ UTR. This may point to different binding modes of *At*GRP7 for different regions of the transcript. Differences in target site recognition in the 3′ UTR versus other regions of its target transcripts have been observed for Musashi1 (MSI1) in human cell culture [68].

Notably, the UCUUCUUC motif located in the 5′ UTR and the exon shows high similarity to two C/U-rich motifs enriched in targets of SR45 that are preferentially found in introns and 5′ UTRs of the SAR transcripts compared to the non-SAR transcripts [5]. A comparison of the SAR genes to the *At*GRP7 targets revealed that 150 of the 858 iCLIP targets and 61 of the high-confidence binders identified by both iCLIP and RIP-seq corresponded to SAR transcripts. Of the RIP-only targets, 503 were also found for SR45. This common set of targets may hint at overlapping or opposing functions of both proteins.

Previously, electrophoretic mobility shift assays revealed binding of *At*GRP7 to the 3′ UTR of its own pre-mRNA. Extensive deletion analysis and mutagenesis of the binding site unraveled a minimal sequence in the 3′ UTR, UUC UGG [39]. This motif was subsequently employed to study the RNA-binding dynamics of *Nt*GR-RBP1, a tobacco orthologue of *At*GRP7, by NMR [69]. Notably, iCLIP identified significant XL sites within a few nucleotides upstream and downstream of this motif, and an enriched pentamer maps to the motif, confirming the in vitro binding data in vivo (Additional file 1: Figure S7a). Furthermore, a binding site within the second half of the intron has been characterized in vitro [14, 18, 40]. Although this region did not return XL sites in the iCLIP studies, an intron motif was detected next to the determined in vitro binding site [40]. It has been observed that in vitro binding studies and in vivo CLIP experiments result in common but also distinct binding sites. For example, for the well-studied *C. elegans* PUF (Pumilio/FBF) protein, iCLIP peaks without the canonical binding element have been identified [33]. It should be noted that, in addition to the sequence context, secondary structure features of the RNA are relevant for binding which are not taken into account in vitro [70]. Furthermore, UV light irradiation in CLIP techniques leads to crosslinking of RNA and protein mainly at uridines and thus some binding sites may be less efficiently identified [71, 72].

The MEME motifs we identified are similar in length to motifs identified for other proteins with a single RRM, e.g. *Arabidopsis* SR45 [5] or hnRNPC1/C2 in HeLa cells [41]. It remains to be determined whether additional proteins that act synergistically or antagonistically with *At*GRP7 may interact with the motifs. Furthermore, the accessory role the glycine-rich stretch has in RNA binding in addition to the RRM suggests that the binding site may be more extended [40].

### Impact of *At*GRP7 on targets

To unravel how *At*GRP7 affects its targets at the RNA level, RNA-seq was performed on plants lacking *At*GRP7 or having an elevated *At*GRP7 level. An advantage of including the *At*GRP7-ox plants is that, in the case of redundancies, changes in the mutant may be masked by other factors, yet upon overexpression a change may be seen. In the *grp7-1 8i* mutant, 4.46% of all DEGs at LL36 (93 of 2087) and 3.28% of all DEGs at LL24 (24 of 731) are iCLIP targets (Additional file 1: Figures S8 and S11); 2.78% of all DEGs at LL36 (58 of 2,087) and 1.92% of all DEGs at LL24 (14 of 731) are high-confidence targets also identified by RIP-seq. This compares well with the findings for the SR-like protein SR45, where 116 out of 4262 SR45 targets identified by RIP-seq (2.72%) are differentially expressed in *sr45-1* [5].

Importantly, our data suggest that the effect of *At*GRP7 on its direct target genes is predominantly repressive (Additional file 1: Figure S12). RNA-seq revealed a similar number of transcripts upregulated or downregulated in *At*GRP7-ox or *grp7-1 8i* plants compared to wt. In stark contrast, the bound targets are enriched for genes expressed at reduced levels in *At*GRP7-ox plants and upregulated in the *grp7-1 8i* mutant. This is seen for the high-confidence binders as well as for iCLIP and RIP-seq targets at both time points.

Among the high confidence binders were a number of transcripts implicated in stress responses. For example, *FAD2*, which is required for salt tolerance, is downregulated in the *At*GRP7-ox plants. It was reported that elevated levels of *At*GRP7 have a negative effect on germination and seedling growth under salt stress conditions [20]. Furthermore, several cold-responsive transcripts were among the binding targets. *At*GRP7 has been shown to promote freezing tolerance [20]. However, no noticeable difference in the expression of several transcripts implicated in freezing tolerance in either the mutant or *At*GRP7-overexpressing plants were found in response to low temperature [20]. It should be noted that numerous cold-responsive transcripts are controlled by the circadian clock and thus upon exposure to low temperature an acute temperature response is overlaid by changes in the circadian oscillator at low temperature [73, 74]. Therefore, more systematic investigations are required to monitor transcriptome changes in plants with altered *At*GRP7 levels across a wider range of low temperatures. It remains possible that the role of *At*GRP7 in freezing tolerance and its impact on the cold-responsive targets occur by another regulatory mechanism not seen at the transcript level. Furthermore, a number of transcripts bound by *At*GRP7 are associated with pathogen defense, including *HSPRO2* and several WRKY transcription factors. Previously, we observed that *At*GRP7 binds to the *PDF1.2* transcript associated with jasmonic acid/ethylene-dependent defense against necrotrophic pathogens and negatively affects its expression, whereas it does not bind to the *PR1* transcript and regulates *PR1* transcription indirectly [36]. Thus, the determination of the *At*GRP7 RNome and the dynamics of posttranscriptional networks controlled by *At*GRP7 in response to pathogens will be revealing.

Although at first sight it seems counterintuitive that a relatively low proportion of the binding targets are differentially regulated at the RNA level, this has been observed before for in vivo binding substrates of both RBPs and transcription factors. A comprehensive iCLIP analysis of the SR protein family in mouse P19 cells revealed that each of the SRSF1 to SRSF7 factors crosslinks to thousands of target transcripts [62]. However, few transcripts are altered in the cytoplasm when individual SRSFs are knocked down, pointing to redundant functions in mRNA export. No strict correlation between binding of *Saccharomyces cerevisiae* Puf3p and altered abundance in *puf3* deletion strains was observed, suggesting that other proteins may contribute to the regulation of the binding targets [75]. Comparison of the 2289 candidate ChIP target genes of the MADS-domain transcription factor APETALA1 (AP1), a key regulator of *Arabidopsis* flower development, with microarray data of the *ap1* mutant revealed 249 genes with > 1.8-fold differential expression [76]. Genome-wide identification of binding sites for two repressors of floral transition, FLOWERING LOCUS C and SHORT VEGETATIVE PHASE, revealed that 15 to 25% of the transcript changes were caused by direct regulation [77]. Furthermore, it should be kept in mind that crosslinking also captures transient interactions and thus may include interactions which are biologically “not meaningful” in the context investigated [78]. This does not rule out, however, that such an interaction may have functional consequences on target RNAs under other circumstances.

### Impact of *At*GRP7 on circadian transcript oscillations

The circadian clock controls transcription of about 30% of the *Arabidopsis* genome [79]. However, transcriptional rhythms lead to oscillations in mRNA steady-state abundance only if an mRNA is sufficiently short-lived [80]. Accordingly, increasing evidence points to regulation at the posttranscriptional level making an important contribution to the circadian transcriptome [81]. So far, it is known that *At*GRP7 negatively autoregulates the oscillations of its own transcript and of the paralog *AtGRP8* through binding to the pre-mRNAs. This causes alternative splicing and subsequent NMD. Mathematical modeling showed that this posttranscriptional regulation in response to rising *At*GRP7 protein levels during the day indeed contributes to the sharp evening peaks of the *AtGRP7* and *AtGRP8* oscillations [82]. Here, we showed that elevated levels of *At*GRP7 dampen circadian oscillations of two of its binding targets, *DRM2* and *CCL*. Furthermore, *AILP1*, previously found to be negatively regulated by *At*GRP7 at the time of its circadian peak, was also a direct in vivo target [18]. Further studies will have to show how *At*GRP7 controls the target genes identified here and how it may exert phase-specific effects that may result from the timing of its binding.

### Effect of *At*GRP7 on RNA processing steps

RNA-seq revealed alternative splicing events significantly changed in the *grp7-1 8i* loss-of-function mutant or *At*GRP7-ox plants. This expands our previous data using a high-resolution RT-PCR based alternative splicing panel [16]. Several of the transcripts that showed changes in alternative splicing upon altered *At*GRP7 levels were identified by iCLIP, RIP-seq, or both. For instance, of the high-confidence binders identified by both approaches, 40 showed changes in alternative splicing in *grp7-1 8i* and 71 in the *At*GRP7-ox plants (Additional file 2: Table S16). A comparison of the splicing patterns of a suite of *At*GRP7 targets at LL24 and LL36 did not reveal a prominent circadian clock-regulated influence of *At*GRP7 on the splicing events.

The overall low number of splicing targets in our analysis may be due to the use of total cell extracts used for the immunoprecipitation, with nuclear RNA thus being less represented. For the genuine *Arabidopsis* splicing factor SR45, 331 of the 4316 RIP targets (7.7%) were differentially spliced in the *sr45-1* mutant [5]. A similar effect was seen for the RBP Musashi1 (MSI1), which is involved in glioblastoma multiforme, the most malignant form of brain cancer. Uren and coworkers identified a large number of binding sites in introns. However, only 26 genes with significant changes in exon usage upon MSI1 knockdown were found, pointing to a rather limited effect of MSI1 on alternative splicing, at least in the cell type studied [68]. In other cases more prevalent effects of an RBP on targets identified by CLIP techniques were found; e.g., 60% of transcripts that are aberrantly spliced upon knockdown of RBM10 are direct targets [83].

Binding targets that are not regulated at the level of steady-state abundance or alternative splicing may be subject to regulation at others steps, e.g., translation or RNA transport. RNA-seq would fail to detect such effects. *At*GRP7 has already been shown to participate in different steps of posttranscriptional control in the cell. This is supported by our finding that *At*GRP7 binds transcripts with introns and without introns. Here we find evidence that *At*GRP7 may also regulate alternative polyadenylation of selected target transcripts. Furthermore, a role for RBPs in translational regulation in the circadian system is proposed by a recent study showing proteins with rhythmic steady-state abundance despite a constant mRNA level [84].

*At*GRP7 has also been shown to function as an RNA chaperone [17]. In line with this, we find transcripts with clusters of crosslink sites, pointing to cooperative binding (Additional file 1: Figure S4b, o). In-depth studies are required to mechanistically connect *At*GRP7 binding to different transcript regions with function.

## Conclusions

Adaptation of iCLIP previously used for UV-irradiated mammalian cell monolayers [10] or *C. elegans* to plant tissue significantly expands the toolkit to identify RNA–protein interactions *in planta*. iCLIP combined with RIP-seq identified a set of 452 high-confidence targets of *At*GRP7, some of which are regulated by *At*GRP7 at the level of steady-state abundance or splicing. Targeted manipulation of the binding motifs detected in the vicinity of the XL sites will reveal their contribution to the control of the targets by *At*GRP7 in the plant. The binding motifs discovered here could now be used to predict additional *At*GRP7 targets. As numerous stress-responsive transcripts were found among the high-confidence binders, a next step is to unravel how posttranscriptional networks controlled by *At*GRP7 are reconfigured under stress.

## Methods

### Plant material

The line *AtGRP7::AtGRP7-GFP* expresses an *At*GRP7-GFP fusion under control of 1.4 kb of the *At*GRP7 promoter and the *At*GRP7 5′ UTR, intron, and 3′ UTR in the *grp7-1* T-DNA mutant, and the line *AtGRP7::GFP* expresses GFP only under control of 1.4 kb of the *At*GRP7 promoter, the *At*GRP7 5′ UTR, and 3′ UTR [16, 85]. The line *AtGRP7::AtGRP7 R* ^*49*^ *Q-GFP* expresses an RNA-binding mutant version with Arg^49^ exchanged for Gln [16]. The *grp7-1 8i* line has an RNAi construct against *At*GRP8 to counteract elevated *AtGRP8* levels due to relief of repression by *At*GRP7 in *grp7-1* [21]. *At*GRP7-ox plants express the *At*GRP7 coding sequence under control of the cauliflower mosaic virus (CaMV) 35S promoter [86].

### Plant growth

*Arabidopsis* seeds were surface-sterilized and sown on half-strength MS (Murashige-Skoog; Duchefa) plates [87]. Plants were grown in 12 h light–12 h dark cycles at 20 °C in Percival incubators (CLF laboratories) followed by free run under continuous light (LL). For RNA analysis, aerial tissue was harvested at subjective dawn or dusk for iCLIP, RIP, and RNA-seq or at 2-h intervals starting 4 h before subjective dawn for qPCR. At least ten plants were bulked for each sample per replicate.

### iCLIP

The iCLIP procedure developed by König and co-workers was adapted to plant tissue [55]. Seedlings on plates were subjected to irradiation with 254-nm UV light at a dose of 500 mJ/cm^2^ in a UVP CL-1000 UV crosslinker on ice. The plant material was quick-frozen in liquid N_2_ and ground to a homogeneous powder with mortar and pestle. Cell lysis buffer (50 mM Tris-HCl, pH 7.5, 150 mM NaCl, 4 mM MgCl_2_, 0.25% Igepal CA-630, 1% SDS, 0.25% sodium deoxycholate, 5 mM DTT, Complete Protease Inhibitor (Roche), 100 U/mL RiboLock (Thermo Fisher), 1 mM phenylmethylsulfonylfluorid) was added to the powder. Lysates were precleared with sepharose beads for 1 h at 4 °C with constant rotation and subjected to immunoprecipitation with GFP Trap beads (Chromotek) or mock precipitation with RFP Trap beads (Chromotek). The RNA–protein complexes were precipitated for 1 h at 4 °C with constant rotation. The beads were washed four times with 1 mL cooled RIP-washing buffer (50 mM Tris-HCl, pH 7.5, 500 mM NaCl, 4 mM MgCl_2_, 0.5% Igepal CA-630, 1% SDS, 0.5% sodium deoxycholate, sodium salt, 2 M urea, 2 mM DTT, Complete Protease Inhibitor) and washed twice with 1 ml cooled original iCLIP wash butter (20 mM Tris-HCl pH 7.4, 10 mM MgCl_2_, 0.2% Tween 20) [10]. On the beads, the precipitate was treated with 2 μl Turbo DNase for 10 min at 37 °C (Thermo Fisher). For RNase digestion, 6.7 U RNase I (Thermo Fisher) were added. For library preparation, the RNAs were dephosphorylated and the L3 linker (Additional file 2: Table S18) was ligated to the 3′ ends using RNA ligase (NEB).

The 5′ termini were labeled using [γ-^32^P] ATP and polynucleotide kinase and the covalently linked RNA–protein complexes were separated on a 4–12% NuPAGE Bis-Tris gel (Thermo Scientific), and electroblotted onto a nitrocellulose membrane. Upon autoradiography, the regions above the fusion protein were cut out and subjected to proteinase K treatment, leaving a polypeptide at the interaction site. Subsequently, RNA was isolated from the membrane using TriReagent and reverse transcribed using primers containing a cleavable adapter region and individual barcode sequences (Additional file 2: Table S18).

After NaOH treatment, the cDNA was purified on a 6% urea-polyacrylamide gel and fragments in the size range of approximately 70–85 nucleotides (high, H), 85–120 nucleotides (medium, M), and 120–200 nucleotides (low, L), respectively, were eluted from the gel (Additional file 1: Figure S2c). The cDNAs were then circularized using CircLigase II (Epicentre) and an oligonucleotide (Cut-oligo) was annealed to generate a BamHI restriction site. Relineariztion via BamHI digestion results in adapters at both ends of the cDNA which were then PCR-amplified. After PCR optimization the three size fractions (H, M, L) were pooled with a ratio of 1:1:1, concentrations were assessed with Qubit dsDNA HS Assay Kit (Thermo Scientific), and 10 nM of the libraries were submitted to high-throughput sequencing after multiplexing of multiple samples. Sequencing was carried out using an Illumina HiSeq2500 (Eurofins) with 50-nucleotide single-end reads or at the Genomics Center of the Max-Planck-Institute for Developmental Biology, Tuebingen, with 100-nucleotide single-end reads.

### RIP-seq

Plants grown in 12 h light–12 h dark cycles for 16 days and subsequently shifted to continuous light were vacuum-infiltrated with 1% formaldehyde for 15 min at LL36 or LL24, followed by quenching with 125 mM glycine. A whole-cell extract was prepared in RIP-lysis buffer (50 mM Tris-HCl pH 7.5, 150 mM NaCl, 4 mM MgCl_2_, 0.25% Igepal CA-630, 1% SDS, 5 mM DTT, 10 mM vanadylribonucleosid complex, 100 U/ml RiboLock (Thermo Fisher), 1 mM phenylmethylsulfonylfluorid, and Complete Protease Inhibitor). The extract was pre-cleared with Sepharose beads and subjected to immunoprecipitation with GFP-Trap beads (Chromotek), hereafter called IP+. After extensive washing with RIP washing buffer (50 mM Tris-HCl pH 7.5, 500 mM NaCl, 4 mM MgCl_2_, 5 mM DTT, 0.5% Igepal CA-630, 1% SDS, 0.5% sodium deoxycholate, 2 M urea), co-precipitated RNAs were eluted with TriReagent and treated with DNase (Promega).

Libraries were prepared from three biological replicates using the Illumina TrueSeq Sample preparation kit, except for omitting the two rounds of poly(A) selection commonly used for total RNA as a starting material. Sequencing was carried out using an Illumina HiSeq2000 at the Genomics Center of the Max-Planck-Institute for Developmental Biology, Tuebingen, with 100-nucleotide single-end reads.

### RNA-seq

Col-2 wt plants, *At*GRP7-ox plants, and the *grp7-1 8i* line were harvested at LL36 or LL24 in parallel to the RIP-seq samples. Total RNA was isolated using the Universal RNA purification Kit (including DNase digestion; EURx, Roboklon). Total RNA integrity was analyzed on an Agilent 2100 Bioanalyzer using the Agilent RNA 6000 Nano Kit and RNA showing a RIN > 8 were further processed. Libraries were prepared from three biological replicates using the TruSeq RNA sample prep kit v2. Sequencing was carried out using an Illumina HiSeq2000 at the Genomics Center of the Max-Planck-Institute for Developmental Biology, Tuebingen, with 100-nucleotide paired-end reads.

### Bioinformatics

#### iCLIP-seq

Raw iCLIP reads were subjected to 3′ adapter trimming and quality filtering using *cutadapt* version 1.9.1 (https://github.com/marcelm/cutadapt). Only reads with a minimal length of 15 nucleotides and a quality score of 20 were kept. The trimmed and filtered reads were de-multiplexed by an in house python script available at https://github.com/GrosseLab/iCLIP. Identical reads including the same random barcode sequence were considered PCR duplicates and hence removed. The barcodes were trimmed from the remaining reads using *barcodeRemoval* from *PIPE-CLIP* [88]. The resulting reads were mapped to the *A. thaliana* TAIR10 reference genome with *STAR* v2.5.2a [89] using the additional transcript annotation file atRTD.gff from atRTD, a novel reference transcriptome containing more than 32,500 additional transcript isoforms [30]. Up to three mismatches were allowed, and only reads mapping uniquely were kept.

Putative XL sites were determined as described previously with minor modifications [10]. XL sites were determined separately for each transcript region due to possible differences in their expression level. For this, introns, exons (concatenated), 5′ UTR, and 3′ UTR were defined as separate regions based on the representative gene model deposited in TAIR10. For the following steps, only the XL sites (−1 position of the reads) were considered, whereas the rest of the reads were omitted.

To determine the significance of each XL site, the FDR was determined. For this, each XL site was extended by 15 nucleotides in both directions and the number of crosslinks were added up for each position, defining a height h. A distribution of the occurrence of each height in the corresponding region was specified, $\left\{{n}_1,{n}_2,\dots {n}_h,\dots {n}_{H-1},{n}_H\right\}$, where $H$ is the maximal height appearing in the region and ${n}_h$ expresses the number of XL sites with height $h$. Therefore, the probability of an observed height of at least $h$ can be expressed by:

$$ P(h)=\frac{\sum_{i=h}^H{n}_i}{N} $$

To discriminate the observed heights from randomly appearing crosslinks, a randomized height distribution was generated 100 times for each region. For this, the same number of $N$ crosslinks were distributed uniformly in the corresponding regions, assembling a background. The mean and standard deviation of each randomly generated height $\left({\mu}_h,{\sigma}_h\right)$ were used to compute the FDR for every observed height:

$$ FDR(h)=\frac{\left({\mu}_h,+,{\sigma}_h\right)}{P(h)} $$

Crosslink positions with a FDR < 0.05 were considered as significant.

To increase reliability, especially in large regions with few crosslinks, the whole significance procedure (assigning XL sites to FDR) was repeated 1000 times to accommodate for variability. Crosslink sites with a FDR < 0.05 in at least 95% of the re-runs were further examined. Only those crosslinks that occurred at the same position in all but one of the replicates were considered for downstream analyses. In datasets with only two replicates, the XLs occurring in both were considered. This computation was performed for the *At*GRP7-GFP samples and the *At*GRP7 R^49^Q-GFP and GFP-only control samples separately. Transcripts with significant crosslink positions in the control samples were removed from the putative *At*GRP7 targets.

#### Motif discovery

A motif search was performed individually on each of the previously defined regions using the MEME suite 4.11.1 [38]. Each XL site was extended by ten nucleotides in either direction. The extraction of genomic sequences was done using bedtools [90], with the addition that thymine (T) occurrences were replaced with uracil (U). The background model was determined by randomly assigning the exact amount of crosslinks uniformly in every region of the transcripts where significant crosslink sites have been identified.

### Determination of pentamer enrichment at XL sites

The Z-score analysis was performed separately for the different transcript regions (introns, exons, 5′ UTRs, and 3′ UTRs). Crosslinks on transcripts antisense to the transcriptional direction of the corresponding gene were removed before proceeding. A random background was generated 100 times for each region, assigning random crosslinks uniformly across the corresponding region. All XL sites (experimental and random) were extended by ten nucleotides in either direction, resulting in 21-nucleotide-long genomic sequences. The pentamer frequency was calculated for each region independently. The Z-score for every pentamer in every region was then determined as follows:

$$ Zscore(P)=\frac{f_{P_{exp}}-\mu {f}_{P_{control}}}{\sigma {f}_{P_{control}}} $$

where *P* is pentamer, *f*_*Pexp*_ is the frequency of the observed pentamer, *μf*_*Pcontrol*_ is the mean frequency of the pentamer in the control dataset, and *σf*_*Pcontrol*_ is the standard deviation of the frequency in the control dataset for the given pentamer.

#### RIP-seq

The evaluation of RIP-seq data was done similarly to [34]. Raw RIP-seq reads were subjected to quality trimming and filtering by *Sickle v1.2* (https://github.com/najoshi/sickle) using parameters -l 50 -q 20. The trimmed and filtered reads were mapped to the *A. thaliana* transcriptome defined by atRTD.gff using *STAR* v2.5.2a [89] with the parameter --quantMode TranscriptomeSAM. Estimated read counts per transcript were obtained by *Salmon* v0.8.2 [91] and summarized into estimated read counts per gene by *tximport* [92]. rRNA, mitochondrial, and chloroplast genes were excluded from the analysis. Transcripts enriched in the RIP samples relative to the RNA-seq samples were detected by *edgeR* as described in the *tximport* vignette (https://github.com/mikelove/tximport/blob/master/vignettes/tximport.md). Genes with a FDR < 0.001 and a log_2_-fold change ≥ 0.5 were considered putative RIP targets.

#### RNA-seq

For the analysis of the RNA-seq data, only the 100-bp R1 reads coming from the original paired-end reads were used for further analysis. Raw RNA-seq reads were processed by the same pipeline as raw RIP-seq reads (see above) with the only exception of *Sickle* parameter –q 30. DEGs between *At*GRP7-ox and wt as well as between *grp7-1 8i* and wt were detected by *edgeR* (see above). Genes with a FDR < 0.05 and a TPM > 1 in at least one of the genotypes were considered putative DEGs.

### Changes in alternative splicing events

The estimated read counts per transcript obtained from Salmon were processed by *tximport* [92]. Alternative splicing events in the atRTD annotation [30] were classified using *SUPPA* [91]. Transcripts having just one annotated isoform were excluded from the analysis. Percentage spliced in (PSI) values, defined as the ratio between the TPM of the isoform including the event and the overall TPM of all isoforms for each splicing event for all genotypes, in wt and *At*GRP7-ox as well as wt and *grp7-1 8i* were compared and tested for significance. Events with a corrected *p* value < 0.01 and |ΔPSI| > 0.1 were considered significant.

### RT-PCR and real-time PCR

For semiquantitative RT-PCR, retrotranscribed RNA was amplified with Taq polymerase in a total volume of 20 μl. To determine the linear range of amplification for each primer pair, samples were withdrawn after 24, 26, 28, 30, 32, and 34 cycles. PCR products were separated on agarose gels and visualized by ethidium-bromide staining. qPCR was performed in a volume of 10 μl with the iTaq SYBR GREEN supermix (Biorad) using 45 cycles of 15 s at 95 °C and 30 s at 60 °C for RIP-qPCR and the circadian RNA kinetics, or in a volume of 20 μl with EvaGreen and GoTaq polymerase (Promega) using 45 cycles of 15 s at 95 °C, 20 s at 55 °C, and 20 s at 72 °C to investigate differentially expressed genes, respectively, in a CFX96 cycler (Biorad). C_q_ values were determined and relative expression levels were calculated based on non-equal efficiencies for each primer pair [93]. Data were normalized to *PP2A* (At1g13320) and expressed as the mean expression levels of the independent biological replicates with two technical replicates each ± standard deviation or as indicated in the figure legend. RIP-qPCR was performed as described [16]. Primers are listed in Additional file 2: Table S18.

### Immunoblot analysis

Immunoblot analysis of lysates and chemiluminescence detection was done as described [94]. Primary antibodies were the antipeptide antibody against *At*GRP7 (rabbit; dilution 1:2500), which discriminates *At*GRP7 from *At*GRP8 and lacks a signal in *grp7-1* [21], a polyclonal serum against LHCP (rabbit; 1:25,000) [95], and a monoclonal antibody against GFP (Roche catalog number 11 814 460 001; mouse; dilution 1:1000). Secondary antibodies were HRP-coupled anti-rabbit IgG (Sigma-Aldrich catalog number A 0545; dilution 1:5000) or HRP-coupled anti-mouse IgG (Sigma-Aldrich catalog number A0168; dilution 1:2500).

## Additional files

Additional file 1: Figure S1. Monitoring for UV stress upon UV crosslinking. Related to Fig. 1. **Figure S2.** iCLIP of *At*GRP7-GFP. **Figure S3.** Significant crosslink sites in *AtGRP7::GFP* (GFP), *AtGRP7::AtGRP7 R* ^*49*^ *Q* -*GFP* (RQ), and *AtGRP7::AtGRP7*-*GFP* samples. **Figure S4.** iCLIP crosslink sites on *At*GRP7 target transcripts. Related to Fig. 3. **Figure S5.** Scatterplot of RIP-seq data versus RNA-seq. **Figure S6.** Clustering of motifs identified by MEME analysis. Related to Fig. 5. **Figure S7.** iCLIP crosslink sites in the *At*GRP7 transcript in comparison to in vitro binding sites. **Figure S8.** Differentially expressed *At*GRP7 targets (DEGs) in plants with altered *At*GRP7 level at LL36. **Figure S9.** Position of the *At*GRP7 crosslink sites within the transcripts at LL24. Related to Fig. 2. **Figure S10.** Candidate binding motifs LL24. Related to Fig. 5. **Figure S11.** *At*GRP7 targets differentially expressed at LL24 in plants with altered *At*GRP7 levels. **Figure S12.** Changes in distribution of the log_2_ fold changes of genes differentially expressed at LL24 (DEGs) in the *grp7-1 8i* mutant or *At*GRP7-ox plants upon binding to *At*GRP7. Related to Fig. 6. **Figure S13.** Validation of differential alternative splicing of *At*GRP7 targets at LL36. **Figure S14.** Validation of iCLIP or RIP-seq alternative splicing targets by RIP RT-PCR. **Figure S15** Validation of differential alternative splicing of *At*GRP7 targets at LL24. (PDF 4700 kb)

Additional file 2: Table S1. Preprocessing of the iCLIP sequencing libraries at LL36 and mapping statistics. **Table S2.** *AtGRP7* iCLIP targets at LL36. **Table S3.** RIP read statistics at LL36. **Table S4.** RIP-seq targets at LL36. **Table S5.** High-confidence binders identified by both iCLIP and RIP-seq at LL36. **Table S6.** Pentamers enriched in the vicinity of the crosslink sites at LL36. **Table S7.** RNA-seq read statistics at LL36. **Table S8.** Differentially expressed *At*GRP7 targets at LL36. **Table S9.** Preprocessing of the iCLIP sequencing libraries at LL24 and mapping statistics. **Table S10.** *At*GRP7 iCLIP targets at LL24. **Table S11.** RIP read statistics at LL24. **Table S12.** RIP-seq targets at LL24. **Table S13.** High-confidence binders identified by both iCLIP and RIP-seq at LL24. **Table S14.** RNA-seq read statistics at LL24. **Table S15.** Differentially expressed *At*GRP7 targets at LL24. **Table S16.** Changes in alternative splicing among *At*GRP7 targets. **Table S17.** Alternative splicing of *At*GRP7 targets at LL24 vs. LL36. **Table S18.** Primers used in this study. (XLSX 888 kb)

## Abbreviations

CLIP: Crosslinking immunoprecipitation
DEG: differentially expressed gene
FDR: False discovery rate
GFP: GREEN FLUORESCENT PROTEIN
iCLIP: Individual nucleotide resolution cross-linking and immunoprecipitation
LL: Continuous light
NMD: Nonsense-mediated decay
PSI: Percent spliced in
PTC: Premature termination codon
RBP: RNA-binding protein
RFP: RED FLUORESCENT PROTEIN
RIP: RNA immunoprecipitation
RNA-seq: high-throughput sequencing of cDNAs
RRM: RNA recognition motif
TPM: Transcripts per million
UTR: Untranslated region
wt: Wild type
XL: Crosslink.
