# Supplementary material for: Adaptation of iCLIP to plants determines the binding landscape of the clock-regulated RNA-binding protein AtGRP7
Source: Genome Biol. 2017 Oct 31;18:204. doi: 10.1186/s13059-017-1332-x (PMC5663106; doi:10.1186/s13059-017-1332-x)
Supplement: Supplementary file 1 — Monitoring for UV stress upon UV crosslinking. Related to Fig. 1. Figure S2. iCLIP of AtGRP7-GFP. Figure S3. Significant crosslink sites in AtGRP7::GFP (GFP), AtGRP7::AtGRP7 R 49 Q -GFP (RQ), and AtGRP7::AtGRP7-GFP samples. Figure S4. iCLIP crosslink sites on AtGRP7 target transcripts. Related to Fig. 3. Figure S5. Scatterplot of RIP-seq data versus RNA-seq. Figure S6. Clustering of motifs identified by MEME analysis. Related to Fig. 5. Figure S7. iCLIP crosslink sites in the AtGRP7 transcript in comparison to in vitro binding sites. Figure S8. Differentially expressed AtGRP7 targets (DEGs) in plants with altered AtGRP7 level at LL36. Figure S9. Position of the AtGRP7 crosslink sites within the transcripts at LL24. Related to Fig. 2. Figure S10. Candidate binding motifs LL24. Related to Fig. 5. Figure S11. AtGRP7 targets differentially expressed at LL24 in plants with altered AtGRP7 levels. Figure S12. Changes in distribution of the log2 fold changes of genes differentially expressed at LL24 (DEGs) in the grp7-1 8i mutant or AtGRP7-ox plants upon binding to AtGRP7. Related to Fig. 6. Figure S13. Validation of differential alternative splicing of AtGRP7 targets at LL36. Figure S14. Validation of iCLIP or RIP-seq alternative splicing targets by RIP RT-PCR. Figure S15 Validation of differential alternative splicing of AtGRP7 targets at LL24. (PDF 4700 kb) [file 13059_2017_1332_MOESM1_ESM.pdf]

Additional file 1: Supplemental Figures 1 - 15

Adaptation of iCLIP to plants determines the binding landscape of the clock-regulated RNA-binding protein *At*GRP7

Katja Meyer[1#], Tino Köster[1#], Christine Nolte[1], Claus Weinholdt[2], Martin Lewinski[1], Ivo Grosse[2,3], Dorothee Staiger[1,*]

[1] RNA Biology and Molecular Physiology, Faculty of Biology, Bielefeld University, Bielefeld, Germany

[2] Institute of Computer Science, Martin-Luther-University Halle-Wittenberg, Halle/Saale, Germany

[3] German Centre for Integrative Biodiversity Research (iDiv) Halle-Jena-Leipzig, Leipzig, Germany

[#] These authors contributed equally to the work

* To whom correspondence should be addressed: dorothee.staiger@uni-bielefeld.de

Figure S1

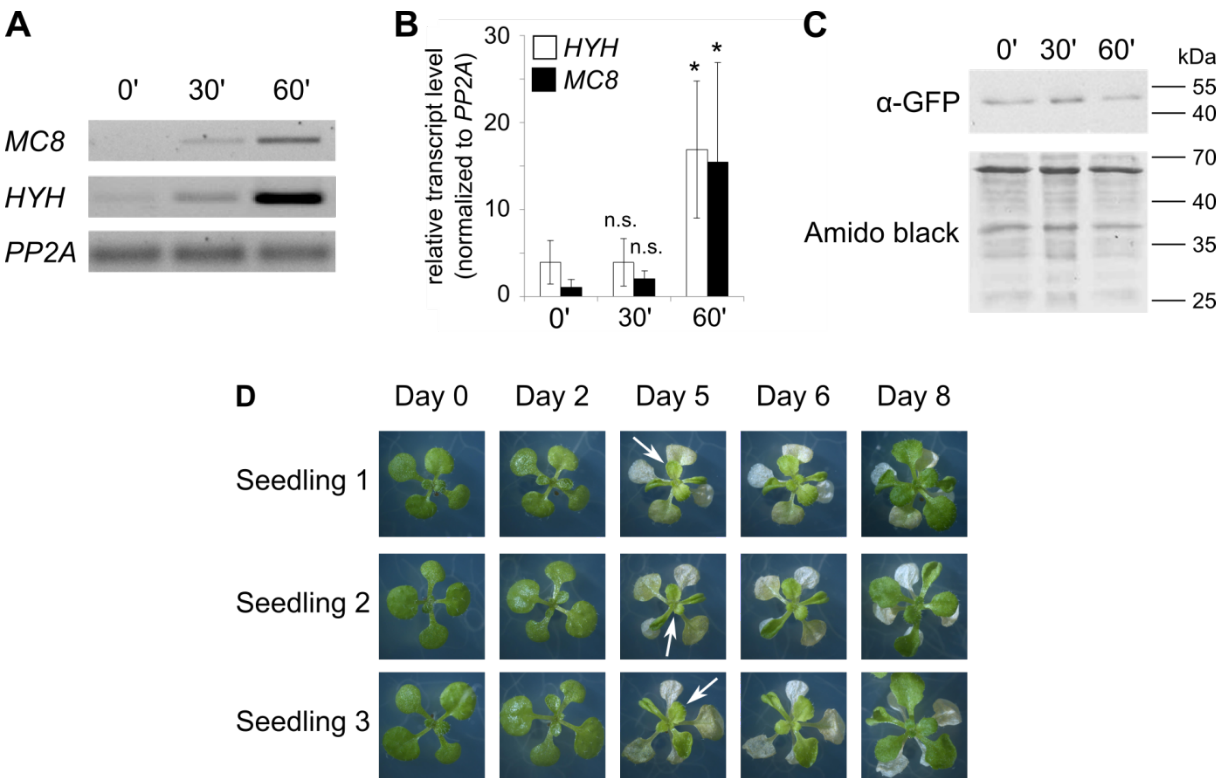

Figure S1 Monitoring for UV stress upon UV crosslinking

Two-week-old seedlings grown in 12 h light- 12 h dark cycles at 20 ^{o}C were exposed to 500 mJ/cm^2 UV-C light (254 nm) and harvested immediately after the irradiation or transferred back to the growth chamber and harvested after 30 min (30’) or 60 min (60’).

The UV marker transcripts *MC8* and *HYH* were detected by RT-PCR (A) and qRT-PCR (B). Significance was determined in three biological replicates by a Mann-Whitney Test (significance indicated by asterisk, $p<0.1$). *PP2A* served as control. n.s., not significant.

C) Immunoblot analysis of *At*GRP7-GFP fusion protein detected with α-GFP antibody (top). Amido black staining of the membrane served as loading control (bottom).

D) Photographs of three representative seedlings before UV crosslinking (day 0) and on days 2, 5, 6, and 8 after UV crosslinking. Arrows indicate newly formed leaves.

Figure S2

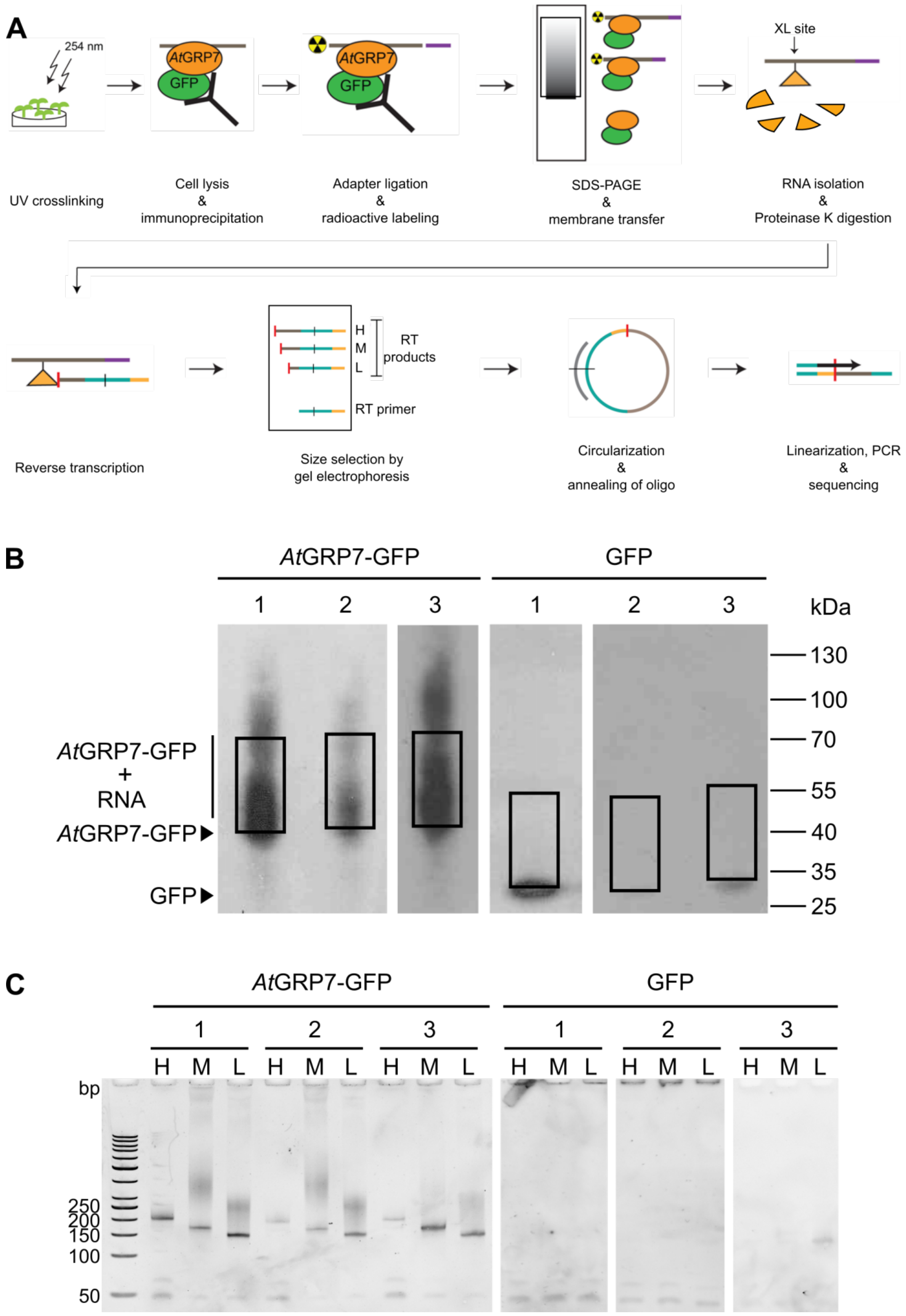
A
254 nm
UV crosslinking
AtGRP7
GFP
Cell lysis & immunoprecipitation
Adapter ligation & radioactive labeling
SDS-PAGE & membrane transfer
XL site
RNA isolation & Proteinase K digestion
Reverse transcription
H
M
L
RT products
RT primer
Size selection by gel electrophoresis
Circularization & annealing of oligo
Linearization, PCR & sequencing
B
AtGRP7-GFP
GFP
1 2 3 1 2 3
kDa
130
100
70
55
40
35
25
AtGRP7-GFP + RNA
AtGRP7-GFP
GFP
C
AtGRP7-GFP
GFP
1 2 3 1 2 3
H M L H M L H M L H M L H M L H M L
bp
250
200
150
100
50

Figure S2 iCLIP of *At*GRP7-GFP

A) Scheme of iCLIP strategy. *Arabidopsis thaliana* seedlings were irradiated with 254 nm UV-C light, the cells were lysed and the protein of interest was immunoprecipitated by its GFP tag. An adapter was ligated to the 3' end of the co-precipitated RNA, whereas the 5' end was radioactively labeled. The precipitated complexes were separated on an SDS-PAGE, transferred onto a membrane and visualized by autoradiography. The RNA was isolated from the membrane and the crosslinked protein was digested by Proteinase K, leaving a short peptide behind. The reverse transcriptase was stopped at this position. cDNAs were size separated, eluted from the gel in three fractions (high H, medium M, low L) and free primers were removed. The cDNAs of the separate fractions were circularized and an oligonucleotide was annealed to generate a restriction site. After linearization, both ends of the cDNA contained adapters. These were used for PCR amplification and high throughput sequencing.

B) Representative autoradiograms of the RNA-protein complexes precipitated from *AtGRP7*::*AtGRP7-GFP grp7-1* and *AtGRP7*::*GFP* plants for three biological replicates. Rectangles indicate the regions that were cut from the membrane. The positions of the molecular weight standards are indicated.

C) cDNAs are amplified by PCR for library preparation. PCR products are visualized on a 6% polyacrylamide gel with the three different sizes (H, M, L) resulting from the previous size selection. A size standard indicates the fragment sizes.

Figure S3

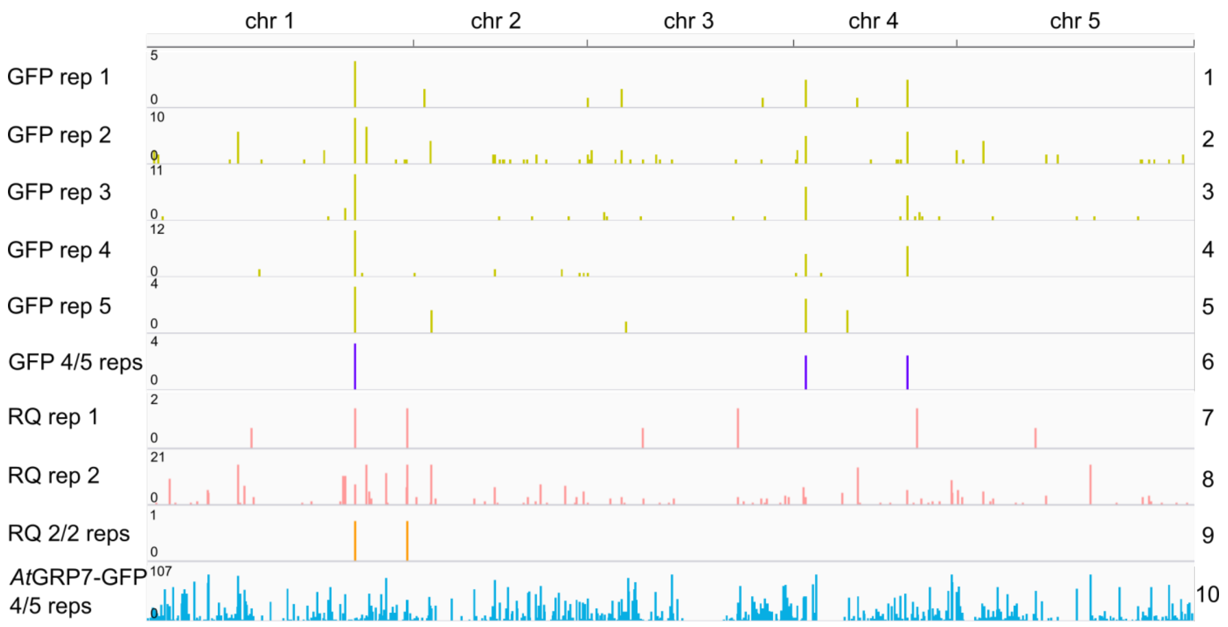

Figure S3 Significant crosslink sites in *AtGRP7::GFP* (GFP), *AtGRP7::AtGRP7 $R^{49}Q$ -GFP* (RQ) and *AtGRP7::AtGRP7-GFP* (*At*GRP7-GFP) samples.

The significant crosslink sites of GFP only plants (panels 1-5 from top) and *AtGRP7::AtGRP7 $R^{49}Q$ -GFP* plants (RQ, panels 7 and 8 from top) distribute evenly across the chromosomes, but only few crosslink sites co-localize in independent replicates of the control samples (panels 6 and 9 from top). The higher density of crosslink sites in the *At*GRP7-GFP samples is shown for comparison (panel 10).

Figure S4

A *COR413-PM1* (AT2G15970)

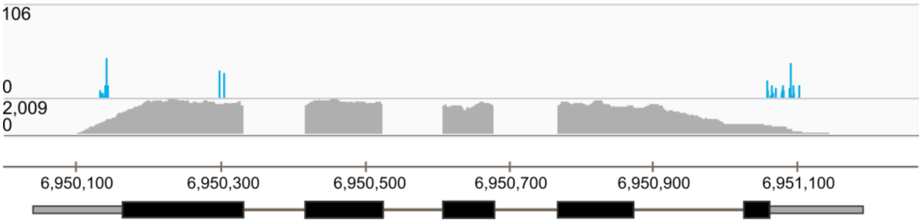

B *PSBP-1* (AT1G06680)

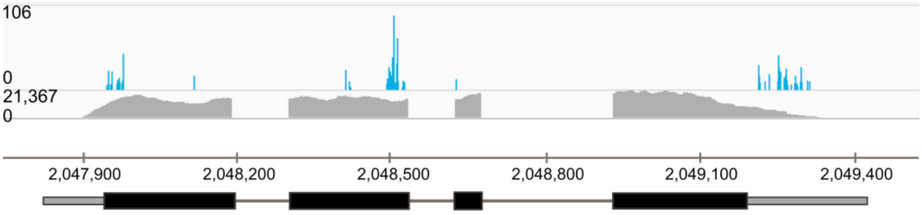

C KH domain-containing protein (AT1G33680)

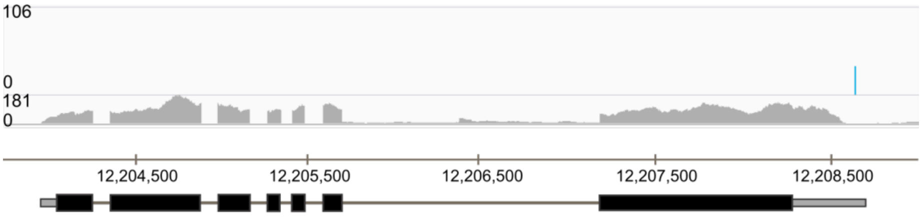

D *THIC* (AT2G29630)

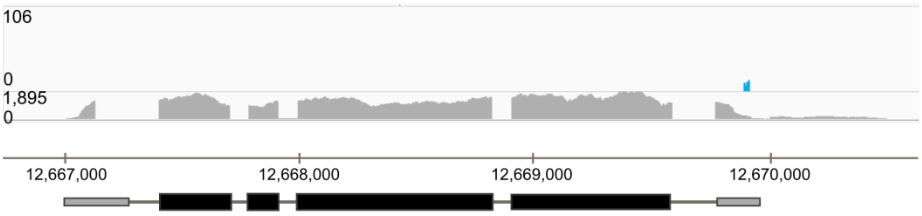

E *COR15A* (AT2G42540)

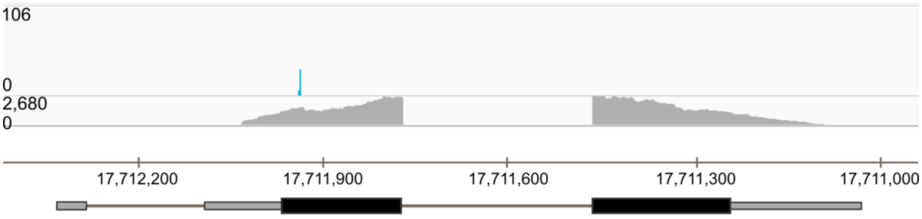

F *COR15B* (AT2G4250)

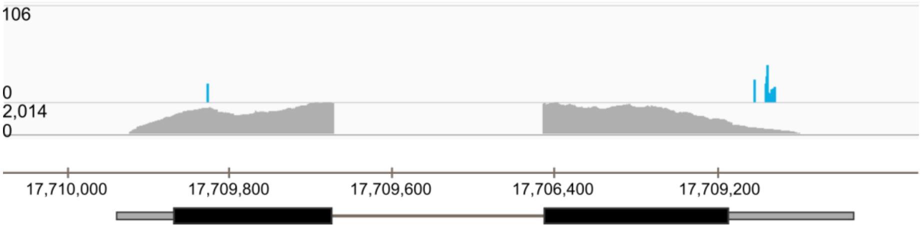

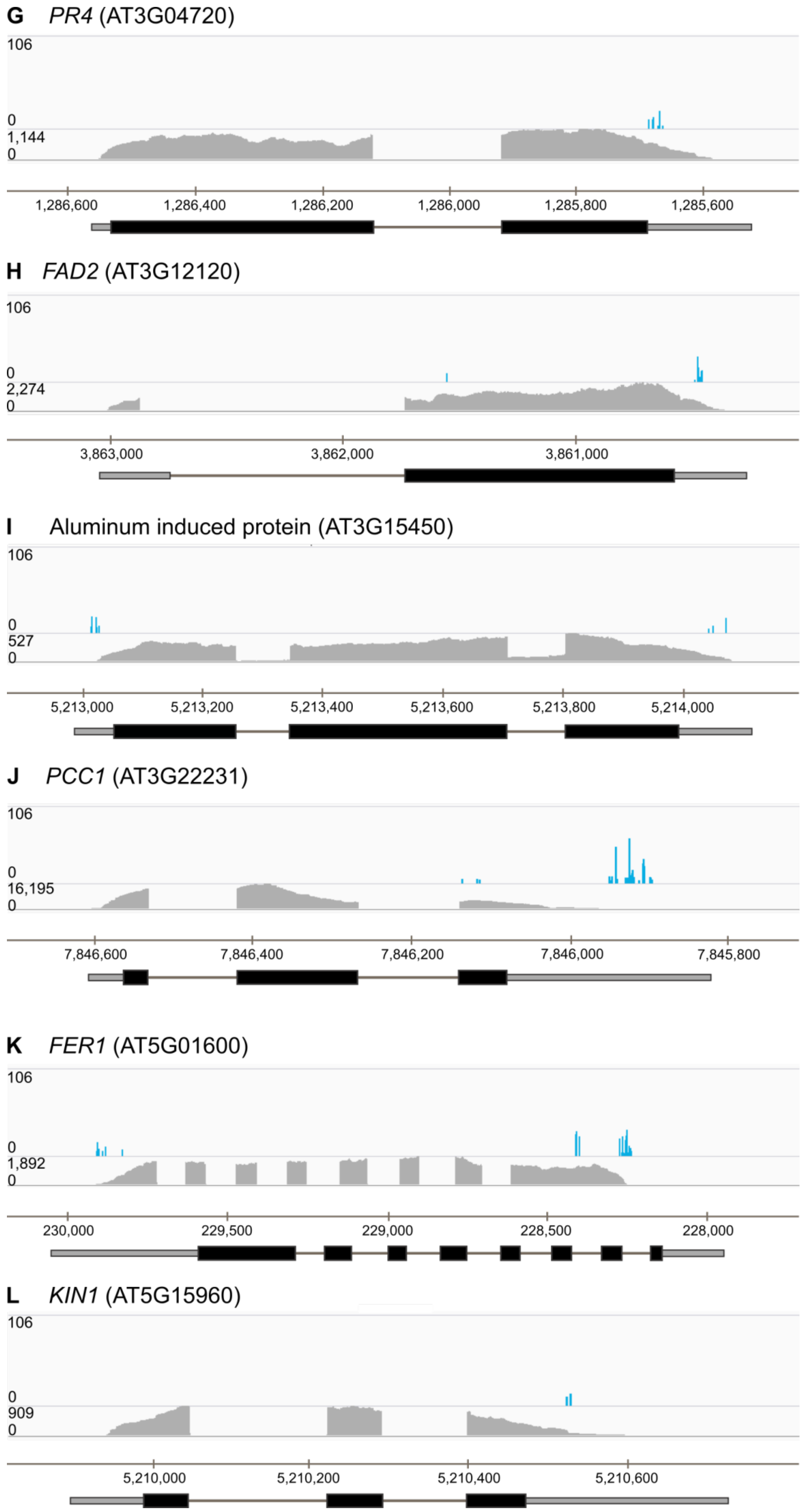
G PR4 (AT3G04720)
106
0
1,144
0
1,286,600
1,286,400
1,286,200
1,286,000
1,285,800
1,285,600
H FAD2 (AT3G12120)
106
0
2,274
0
3,863,000
3,862,000
3,861,000
I Aluminum induced protein (AT3G15450)
106
0
527
0
5,213,000
5,213,200
5,213,400
5,213,600
5,213,800
5,214,000
J PCC1 (AT3G22231)
106
0
16,195
0
7,846,600
7,846,400
7,846,200
7,846,000
7,845,800
K FER1 (AT5G01600)
106
0
1,892
0
230,000
229,500
229,000
228,500
228,000
L KIN1 (AT5G15960)
106
0
909
0
5,210,000
5,210,200
5,210,400
5,210,600

M *COR27* (AT5G42900)

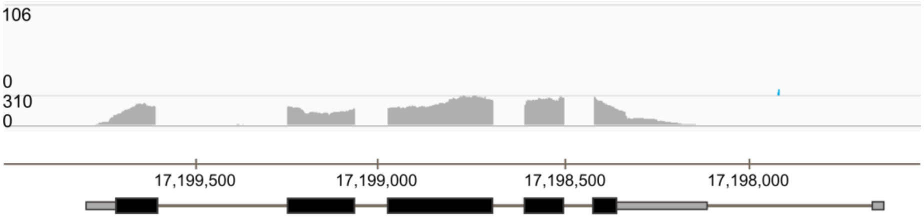

N *RD29A* (AT5G52310)

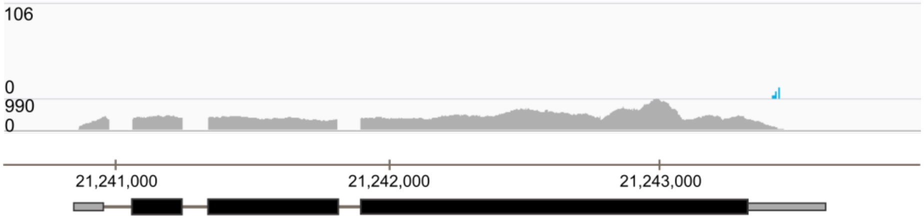

O *ATPC1* (AT4G04640)

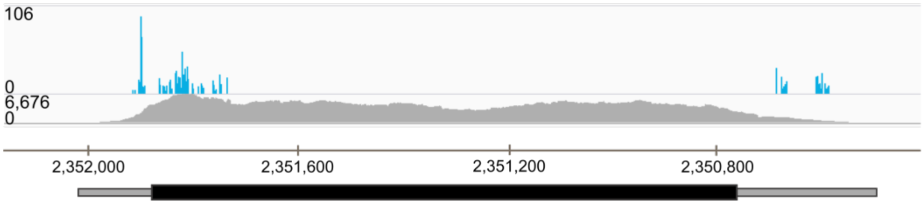

Figure S4 iCLIP crosslink sites on *At*GRP7 target transcripts

Each panel shows the IGV genome browser tracks of significant crosslink sites determined in four out of five biological replicates of *AtGRP7::AtGRP7-GFP grp7-1* plants at LL36 (top), the read counts in the LL36 RNA-seq (mean of three biological replicates; middle), and the representative gene model and chromosomal position (bottom). Thin bars represent 5'UTR (left) and 3'UTR (right); thick bars denote exons and lines denote introns.

A) *COR413-PM1* shows significant crosslink sites in exon 1 and both UTRs.

B) *PSBP-1* shows significant crosslink sites in UTRs and exons.

C) The KH domain-containing protein (AT1G33680) has a significant crosslink site in the 3' UTR.

D) *THIC* shows significant crosslink sites in the 3'UTR.

E) *COR15A* shows significant crosslink sites in exon 1.

F) *COR15B* shows significant crosslink sites in exon 1 and the 3'UTR.

G) *PR4* shows significant crosslink sites in the 3'UTR.

H) *FAD2* shows significant crosslink sites in its exon and its 3'UTRs.

I) Aluminum induced protein (AT3G22231) shows significant crosslink sites in both UTRs.

J) *PCC1* (AT3G22231) shows significant crosslink sites in the last exon and the 3'UTR.

K) *FER1* shows significant crosslink sites in the 5'UTR, exons and introns.

L) *KIN1* shows significant crosslink sites in the 3'UTR.

M) *COR27* shows significant crosslink sites its last intron.

N) *RD29A* shows significant crosslink sites in the 3'UTR.

O) *ATPC1* shows significant crosslink sites in both UTRs and the exon.

Figure S5

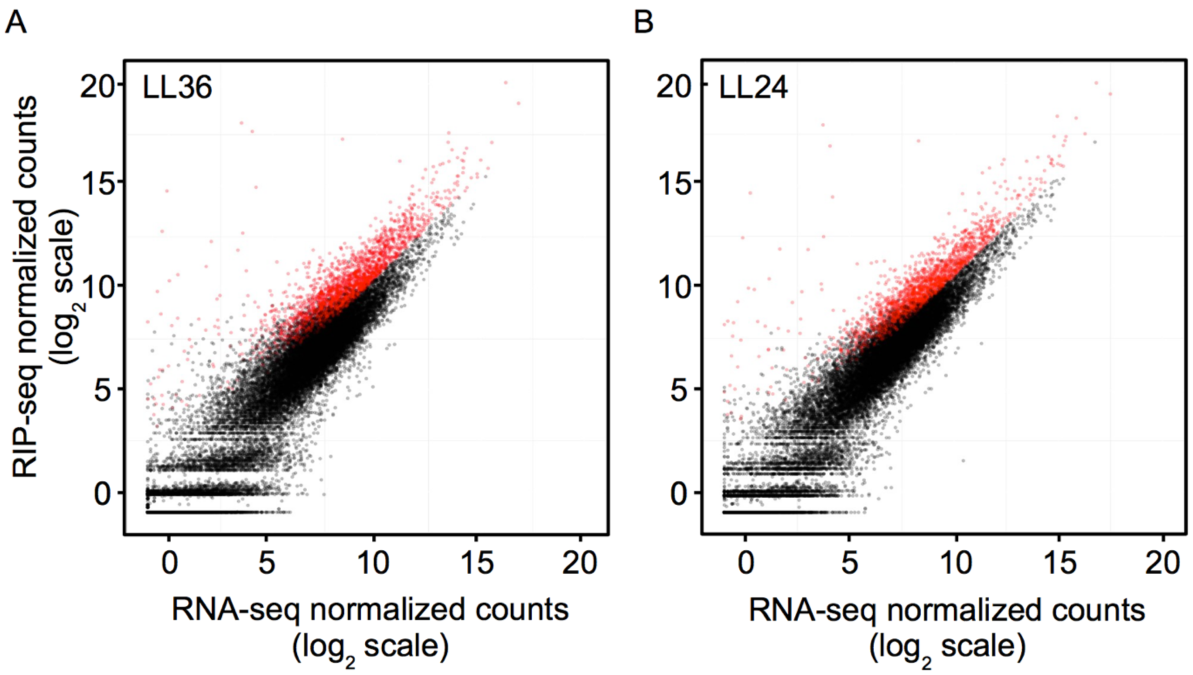

Figure S5 Scatterplot of RIP-seq data *versus* RNA-seq

Normalized mean read counts (trimmed mean of M values, TMM) for each transcript detected in RIP-seq samples of *AtGRP7::AtGRP7-GFP grp7-1* plants were plotted against the read counts (TMM) for the same transcript in the RNA-seq samples from Col-2 wild type plants. Red dots represent significantly enriched transcripts with a $\log_2$ fold change >0.5 (q<0.001). Black dots represent transcripts with a $\log_2$ fold change <0.5 (q<0.001) or with a $\log_2$ fold change >0.5 but not significantly enriched (q>0.001). All transcripts with a TPM<5 in the RIP-seq data were excluded from analysis.

A) Scatterplot of RIP-seq data for time point LL36.

B) Scatterplot of RIP-seq data for time point LL24

Figure S6

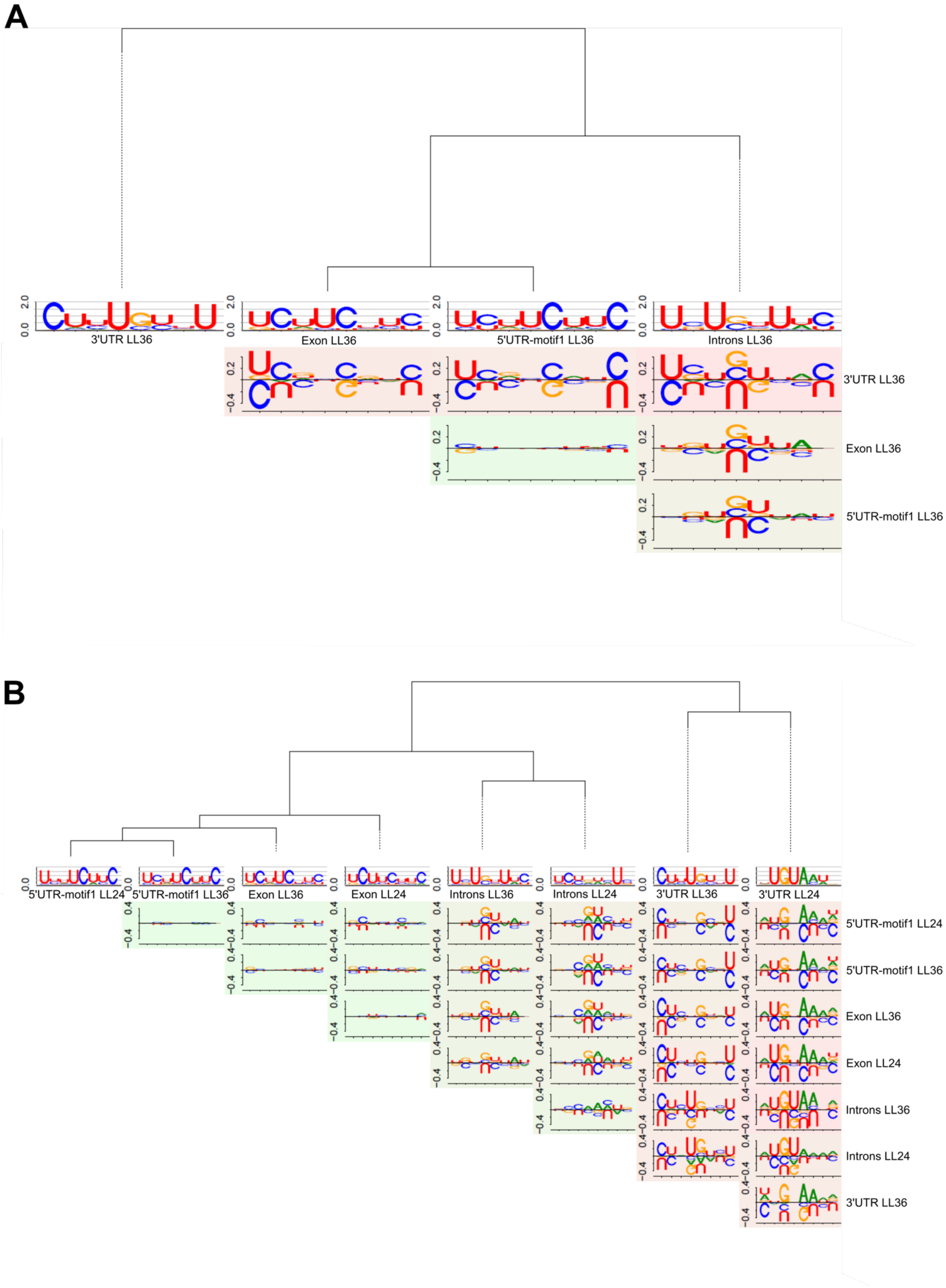
A
3'UTR LL36
Exon LL36
5'UTR-motif1 LL36
Introns LL36
3'UTR LL36
Exon LL36
5'UTR-motif1 LL36
B
5'UTR-motif1 LL24
5'UTR-motif1 LL36
Exon LL36
Exon LL24
Introns LL36
Introns LL24
3'UTR LL36
3'UTR LL24
5'UTR-motif1 LL24
5'UTR-motif1 LL36
Exon LL36
Exon LL24
Introns LL36
Introns LL24
3'UTR LL36

Figure S6 Clustering of motifs identified by MEME analysis

Comparison of binding motifs identified at LL36 (A) and identified both time points LL24 and LL36 (B) using the R package *DiffLogo* [1]. The distance tree was computed using hierarchical clustering and the pair-wise Jensen-Shannon divergence between the motifs. Similar motifs appear closer in the tree (e.g. exon and 5'UTR motif in A). Distances between each motif are displayed using the background color from green (similar) to red (dissimilar) in the difference logo below each tree. Dissimilarities between the position of each single nucleotide are represented by the increasing stack height.

Figure S7

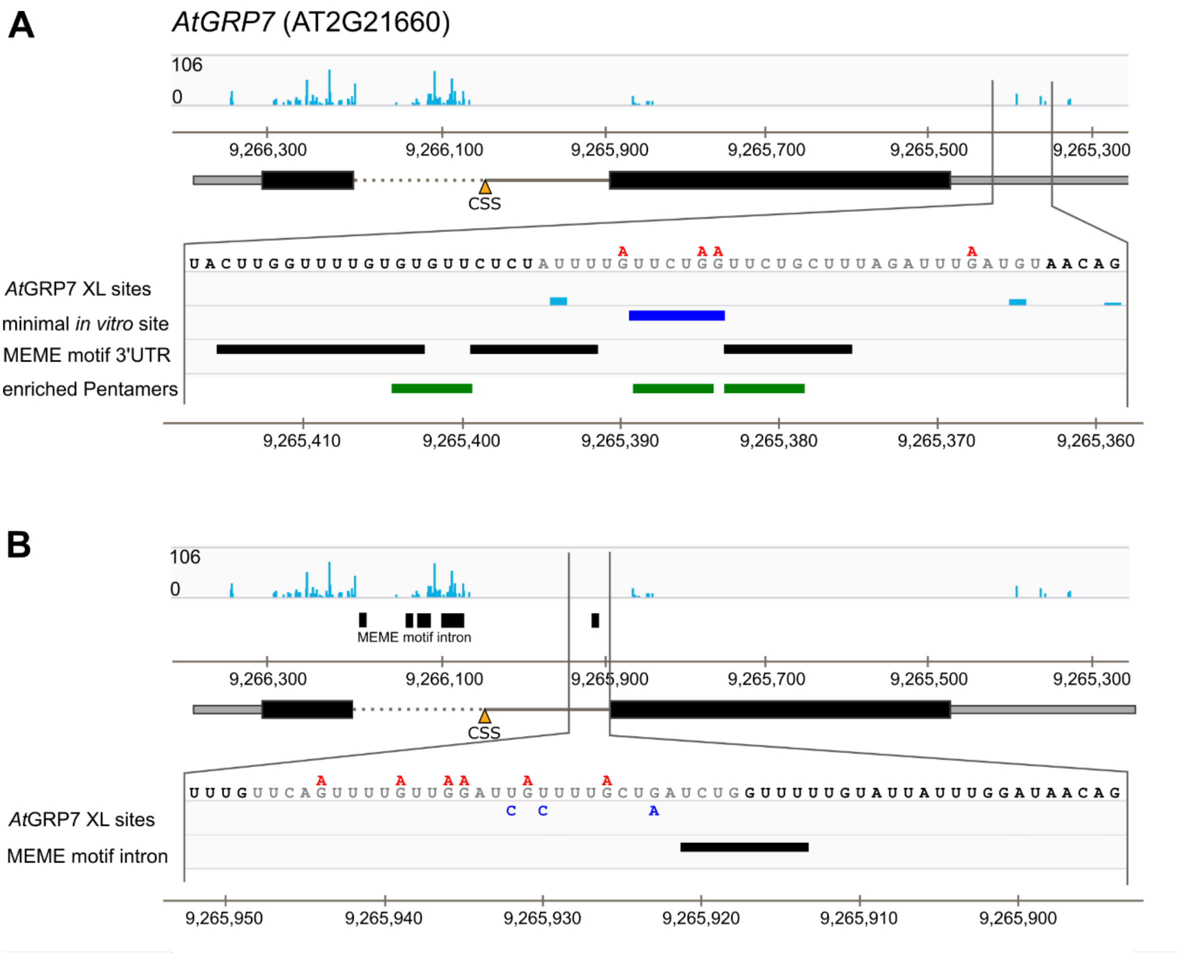

Figure S7 iCLIP crosslink sites in the *At*GRP7 transcript in comparison to *in vitro* binding sites.

The IGV genome browser track shows the significant crosslink sites determined in four out of five biological replicates of *AtGRP7::AtGRP7-GFP grp7-1* plants at LL36 (top panels in A and B). Thin bars represent 5’UTR (left) and 3’UTR (right); thick bars denote exons and lines denote introns.

A) Binding site in the *AtGRP7* 3´UTR.

The area enlarged below the gene model shows a close-up of the crosslink sites in the 3’UTR with the corresponding nucleotide sequence.

Grey nucleotides represent the synthetic oligonucleotide 7-UTR_WT used as binding substrate for recombinant *At*GRP7 in electrophoretic mobility shift assays [2]. Red nucleotides represent point mutations in the corresponding oligonucleotide 7-UTR_G_4mut that impaired *in vitro* binding of recombinant *At*GRP7 in electrophoretic mobility shift assays [2]. Light blue boxes correspond to significant crosslink sites identified in this study. The dark blue box represents a minimal binding sequence for recombinant *At*GRP7 determined by

fluorescence correlation spectroscopy [3]. The black boxes represent the significant MEME 3´UTR motifs at LL36 (identified using the FIMO tool from the MEME suite; cf. Additional file 1: Fig. S6). The green boxes represent the enriched pentamers (cf. Table S6).

B) Binding site in the *AtGRP7* intron.

The broken line indicated the first half of the intron, upstream of the cryptic 5´splice site (CSS).

The area enlarged below the gene model shows a close-up of the crosslink sites in the intron with the corresponding nucleotide sequence.

Grey nucleotides represent the synthetic oligonucleotide 7-intron_WT used as binding substrate for recombinant *At*GRP7 in electrophoretic mobility shift assays [2]. Red nucleotides represent point mutations in the corresponding oligonucleotide 7-intron_G_6mut that impaired *in vitro* binding of recombinant *At*GRP7 [2]. Blue nucleotides correspond to mutations in the synthetic oligonucleotides 7-intron_mut4, 7-intron_mut6, and 7-intron_mut13 that impaired binding of recombinant *At*GRP7 in fluorescence correlation spectroscopy [4]. Light blue boxes correspond to significant crosslink sites identified in this study. The black boxes represent the significant MEME intron motif at LL36 (identified using the FIMO tool from the MEME suite; cf. Additional file 1: Fig. S6).

Figure S8

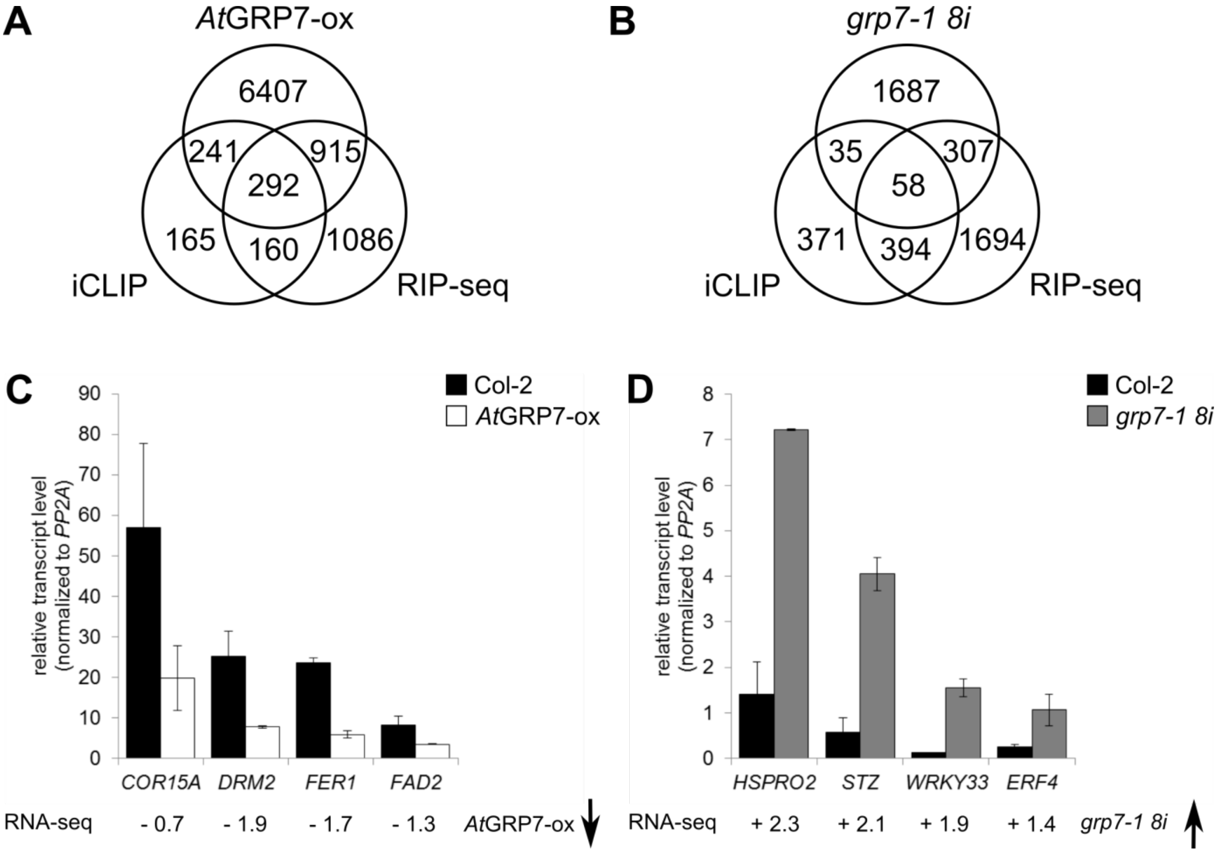

Figure S8 Differentially expressed *At*GRP7 targets (DEGs) in plants with altered *At*GRP7 level at LL36.

The Venn diagrams show significantly differentially expressed genes with q<0.05 in *At*GRP7-ox plants (A) or the *grp7-1 8i* mutant (B) for the targets identified by iCLIP, RIP-seq, or the high-confidence binders identified by iCLIP and RIP-seq with a TPM>1 in at least one of the lines.

C) Validation of DEGs in the *At*GRP7-ox plants.

D) Validation of DEGs in *grp7-1 8i* mutant.

C) and D) show the mean of two biological replicates with two technical replicates each. Numbers below the plot indicate the log_2 fold changes of *At*GRP7-ox versus Col-2 wild type or *grp7-1 8i* versus Col-2 wild type, respectively, obtained in RNA-seq at LL36.

Figure S9

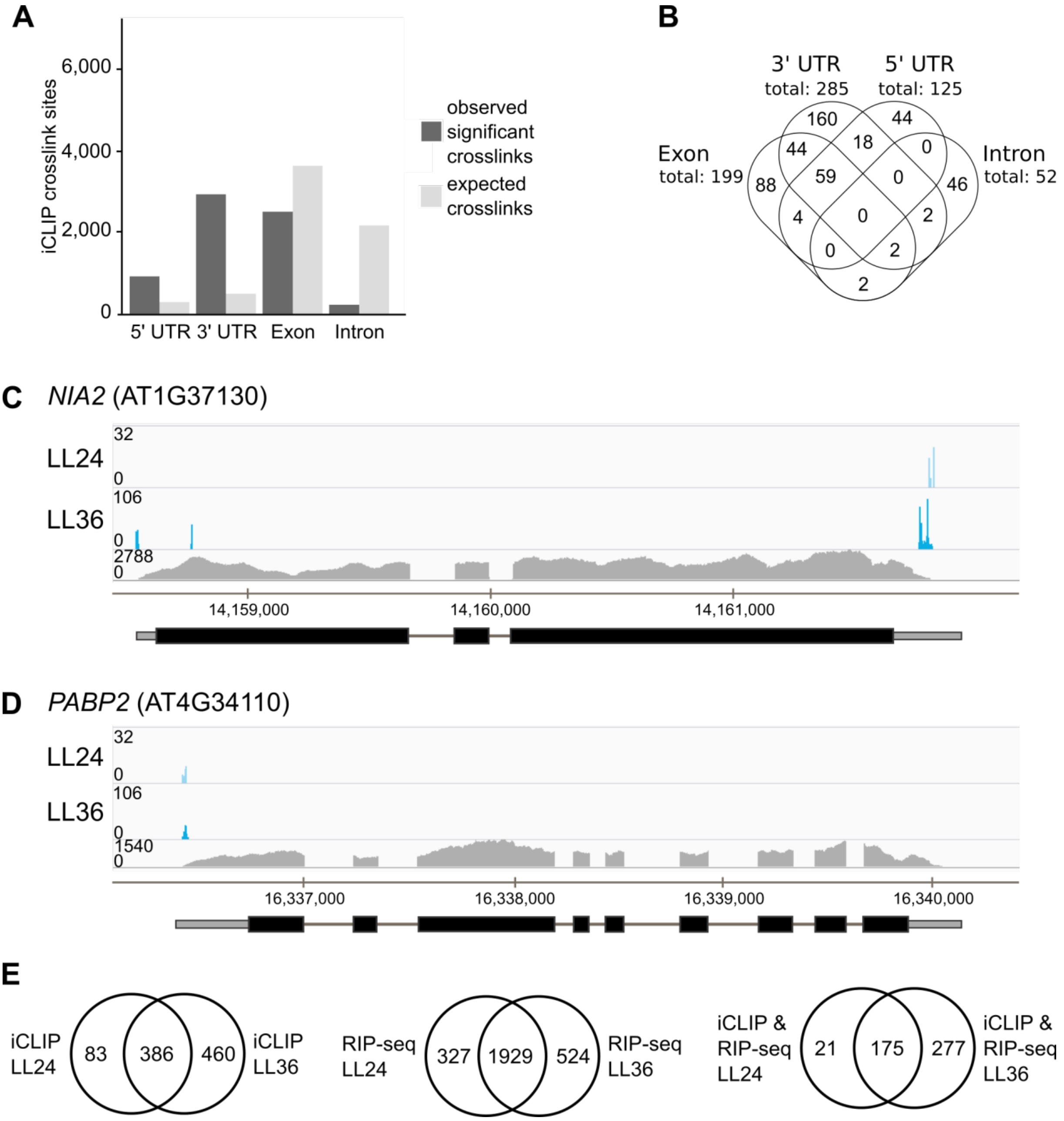

Figure S9 Position of the *At*GRP7 crosslink sites within the transcripts at LL24.

A) Number of significant crosslink sites (FDR<0.05) in different transcript regions normalized to feature length in comparison to an unbiased expectation. In all of the regions a significant difference (*p*<0.001, hypergeometric) could be observed.

B) Distribution of the crosslink sites between the different transcript regions. Numbers outside the Venn diagram (“total”) state the overall number of transcripts with crosslink sites in the respective regions.

C+D) Each panel shows the IGV genome browser tracks of significant crosslink sites determined in four out of five biological replicates of *AtGRP7::AtGRP7-GFP grp7-1* plants at LL36 (1st row) and two out of three biological replicates of *AtGRP7::AtGRP7-GFP grp7-1* plants at LL24 (2nd row), the read counts in the LL36 RNA-seq (mean of three biological replicates; 3rd row), and the representative gene model and chromosomal position (bottom row) of *NIA2* (C) and *PABP2* (D). Thin bars represent 5'UTR (left) and 3'UTR (right); thick bars denote exons and lines denote introns.

E) Venn diagrams illustrate the overlap between the two time points for the three data sets iCLIP, RIP-seq and the high-confidence binders identified by both iCLIP and RIP-seq.

Figure S10

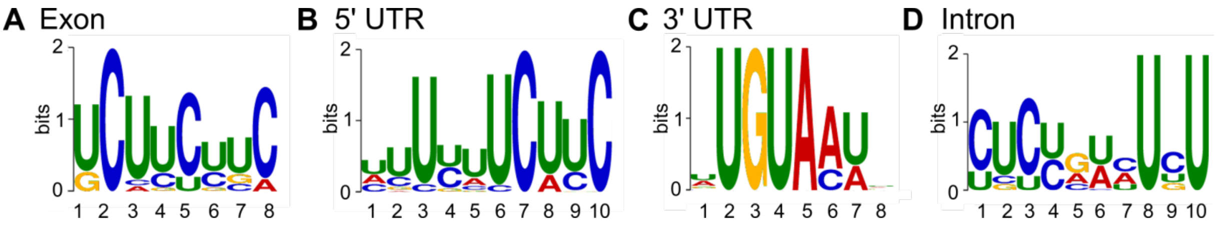

Figure S10 Candidate binding motifs LL24
Most significant motifs (based on their p value) identified by MEME analysis of the 21-nt region around the significant crosslink sites (FDR<0.05) occurring in at least two out of three replicates in A) exons, B) 5’UTR, C) 3’UTR and D) intron of binding targets at LL24.

Figure S11

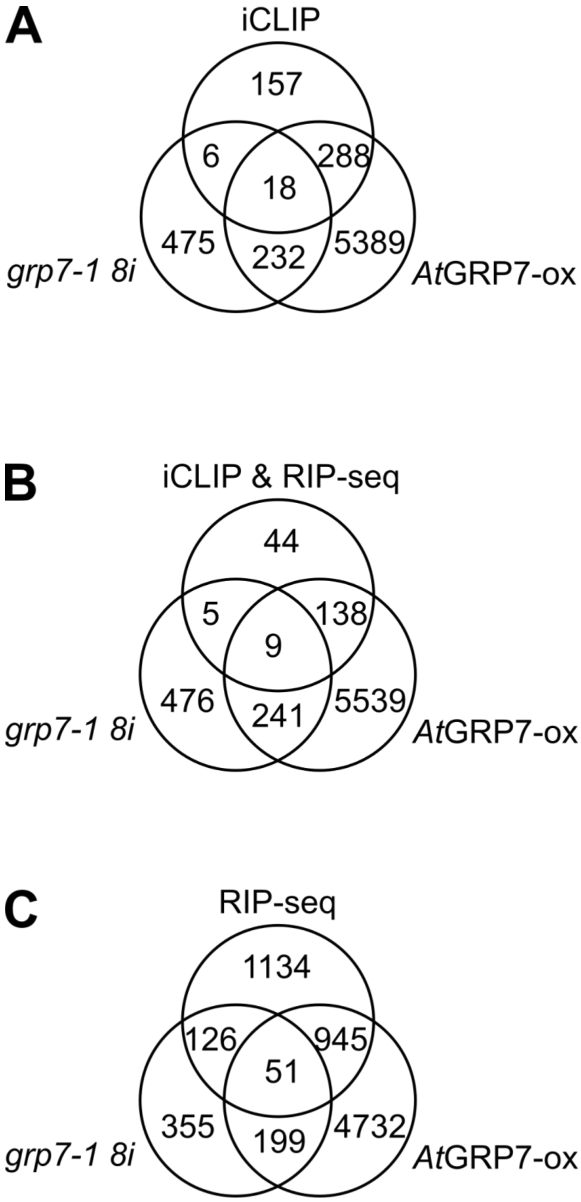

Figure S11 *At*GRP7 targets differentially expressed at LL24 in plants with altered *At*GRP7 levels.

The Venn diagrams show significantly differentially expressed genes with q<0.05 (DEGs) in the *grp7-1 8i* mutant or *At*GRP7-ox plants for A) the targets identified by iCLIP, B) the high-confidence binders identified by iCLIP and RIP-seq, and C) all targets identified by RIP-seq.

Figure S12

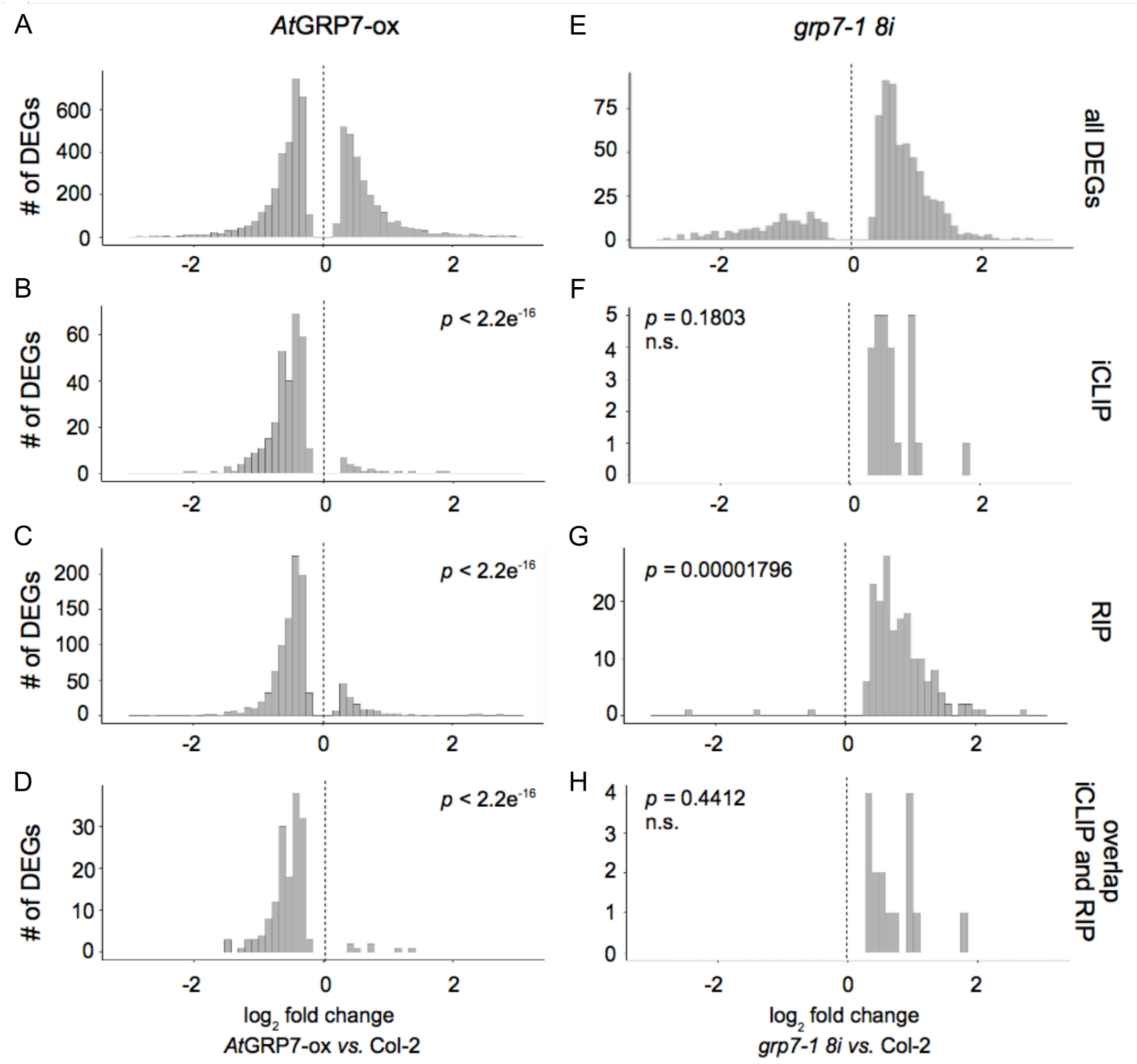

Figure S12 Changes in distribution of the log_2 fold changes of genes differentially expressed at LL24 (DEGs) in the *grp7-1 8i* mutant or *At*GRP7-ox plants upon binding to *At*GRP7. Log_2 fold change distribution of all differentially expressed genes (DEGs) from the RNA-seq data set (A, *At*GRP7-ox, E *grp7-1 8i*), as well as of iCLIP targets with a significant differential expression (B, *At*GRP7-ox, F *grp7-1 8i*), RIP targets with a significant differential expression (C, *At*GRP7-ox, G *grp7-1 8i*) and high-confidence binders identified by both iCLIP and RIP (D, *At*GRP7-ox, H *grp7-1 8i*). The distribution of all identified DEGs in RNA-seq (A, E) was tested pairwise against all target groups. The resulting *p*-value is displayed accordingly.

Figure S13

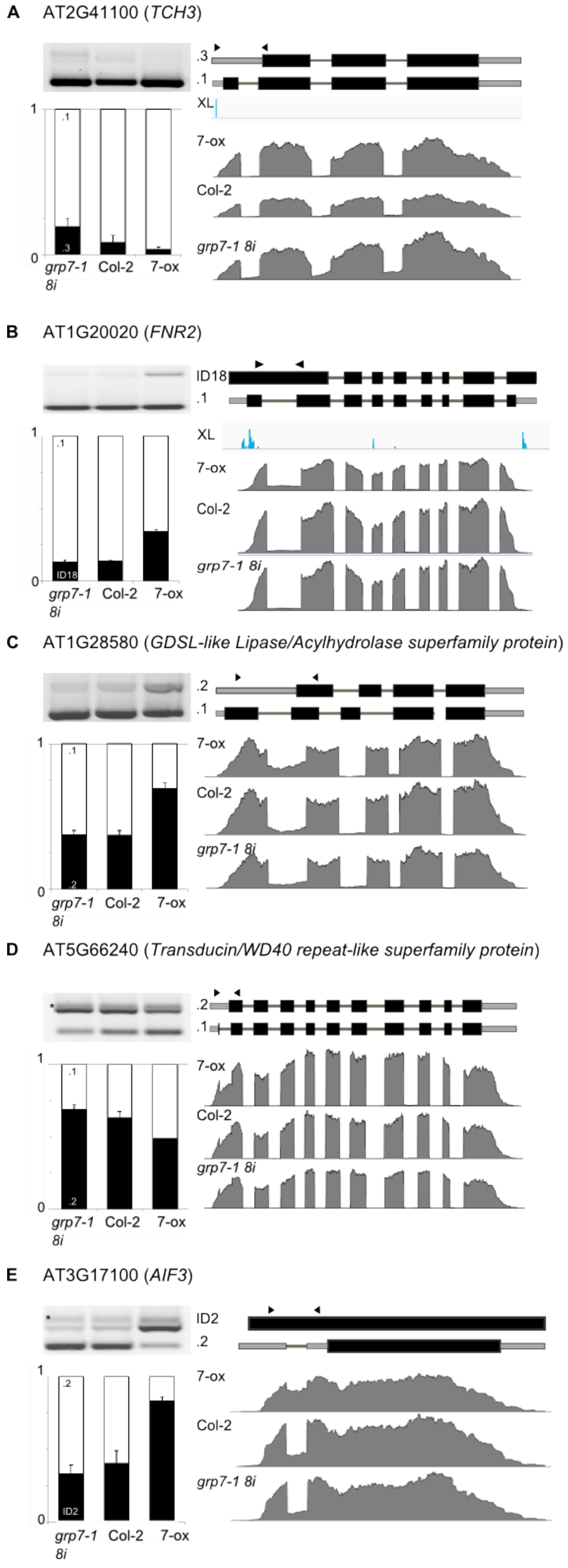
A AT2G41100 (*TCH3*)
.3
.1
XL
7-ox
Col-2
grp7-1 8i
1
0
.1
.3
grp7-1 8i
Col-2
7-ox
B AT1G20020 (*FNR2*)
ID18
.1
XL
7-ox
Col-2
grp7-1 8i
1
0
.1
ID18
grp7-1 8i
Col-2
7-ox
C AT1G28580 (*GDSL-like Lipase/Acylhydrolase superfamily protein*)
.2
.1
7-ox
Col-2
grp7-1 8i
1
0
.1
.2
grp7-1 8i
Col-2
7-ox
D AT5G66240 (*Transducin/WD40 repeat-like superfamily protein*)
.2
.1
7-ox
Col-2
grp7-1 8i
1
0
.1
.2
grp7-1 8i
Col-2
7-ox
E AT3G17100 (*AIF3*)
ID2
.2
7-ox
Col-2
grp7-1 8i
1
0
.2
ID2
grp7-1 8i
Col-2
7-ox

F AT1G33265 (*FAX4*)

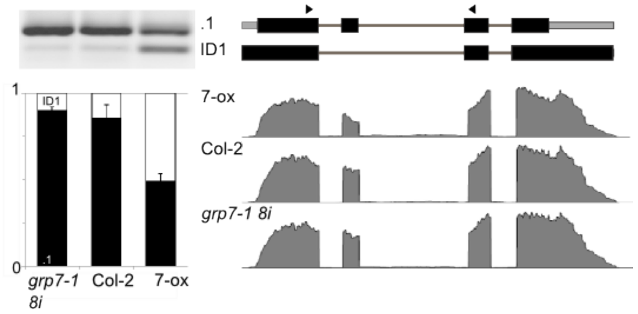

G AT1G76020 (*Thioredoxin superfamily protein*)

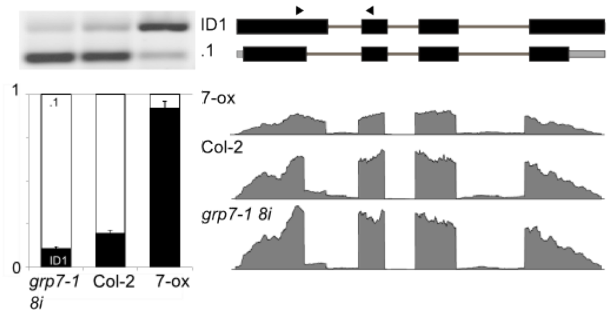

H AT1G45474 (*LHCA5*)

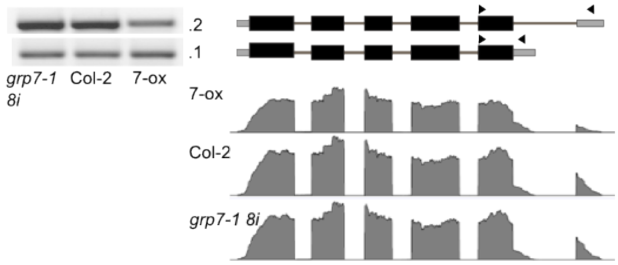

I AT2G29630 (*THIC*)

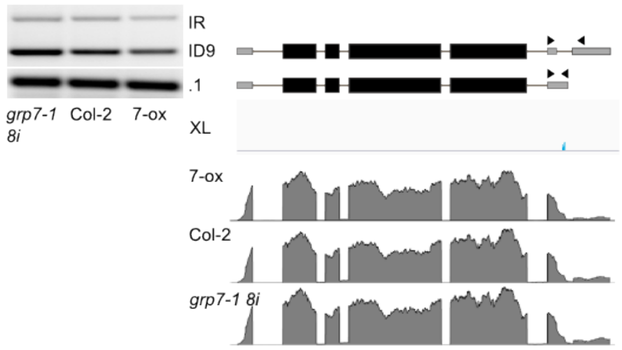

Figure S13 Validation of differential alternative splicing of *At*GRP7 targets at LL36
Each panel depicts a representative RT-PCR result of the alternative splicing event (top left), the gene model of the representative isoforms based on the atRTD annotation [5] (top right), a bar plot indicating the expected relative amount of the isoform calculated from the RNA-seq data (bottom left) and an RNA coverage plot visualizing the read coverage (bottom right).

TCH3 (A) shows a significantly increased intron retention in *grp7-1 8i* compared to the wild type. *FNR2* (B) shows enhanced intron retention in *At*GRP7-ox. A GDSL-Like Lipase/Acylhydrolase superfamily protein (C) also retains intron 1 more often in *At*GRP7-ox than in the wild type. A Transducin/WD40 repeat-like superfamily protein (D) shows a higher amount of the intron-spliced isoform in *At*GRP7-ox than in the wild type. *AIF3* (E) shows higher levels of intron retention in *At*GRP7-ox than in the wild type. An exon of *FAX4* (F) is skipped more often in *At*GRP7-ox than in the wild type. A thioredoxin superfamily protein (G) shows a differential usage of an alternative 5' splice site in *At*GRP7-ox. In *LHCA5* (H) and *THIC* (I) alternative splicing leads to an alternative polyadenylation site. In both cases the distal polyadenylation site is reduced in *At*GRP7-ox.
Events were validated by RT-PCR in two independent biological replicates taken at LL36, shown are representative results from *grp7-1 8i*, Col-2 and *At*GRP7-ox (7-ox) samples (left to right). Asterisks indicate irrelevant by-products. Gene models are shown for the relevant isoforms with thin bars representing the 5'UTR (left) and 3'UTR (right), thick bars denoting exons and lines depicting introns. Arrows indicate the position of the PCR primers. The bar plots represent the expected relative amount of the indicated isoform calculated from the RNA-seq data using SUPPA in three independent biological replicates. The RNA coverage plots visualize the read coverage for the respective samples at LL36 and crosslink positions (XL), if available. Information on the transcripts is taken from TAIR 10 and ARAPORT [6].

Figure S14

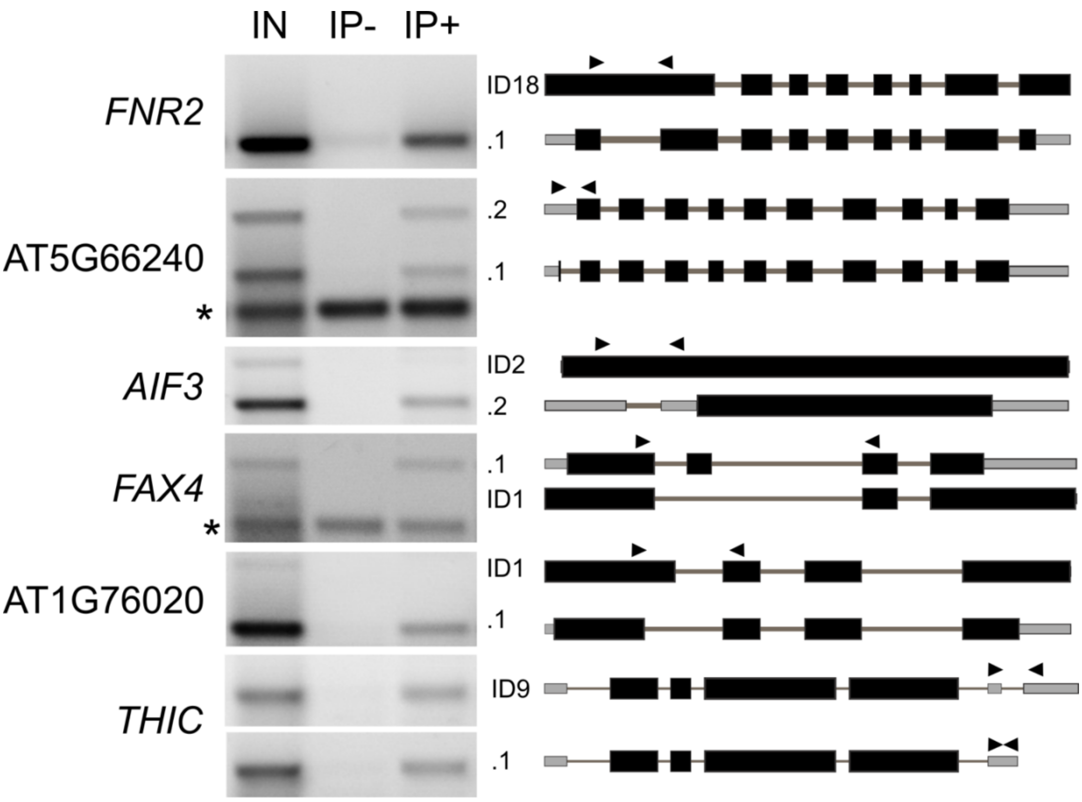

Figure S14 Validation of iCLIP or RIP-seq targets alternative splicing targets by RIP RT-PCR.

Representative RT-PCRs show that one or both isoforms were found to be precipitated by *At*GRP7-GFP (IP+), but not in the mock precipitation (IP-). IN, input. The gene model based on atRTD annotation [5] is indicated for each relevant isoform. Thin grey boxes denote 5´UTR (left) and 3´UTR (right), thick black boxes denote exons, and thin lines denote introns. The positions of the primers are indicated by arrowheads.

Similar results were found in three independent biological replicates. Asterisks indicate irrelevant by-products.

Figure S15

A AT2G41100 (*TCH3*)

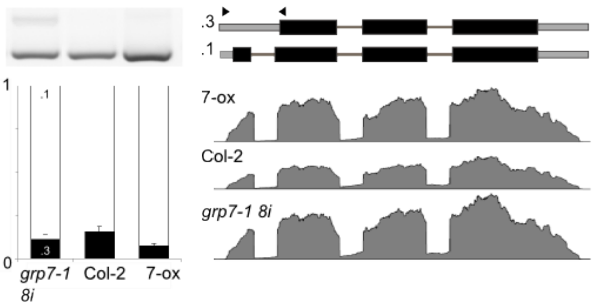

B AT1G20020 (*FNR2*)

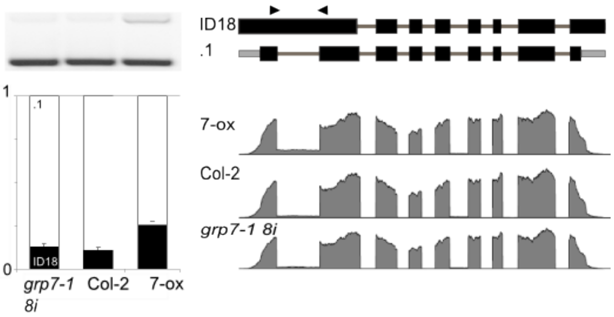

C AT1G28580 (*GDSL-like Lipase/Acylhydrolase superfamily protein*)

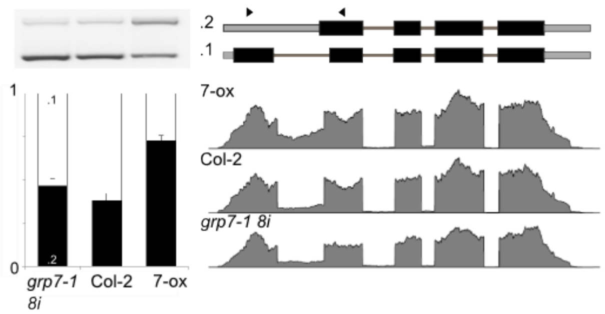

D AT5G66240 (*Transducin/WD40 repeat-like superfamily protein*)

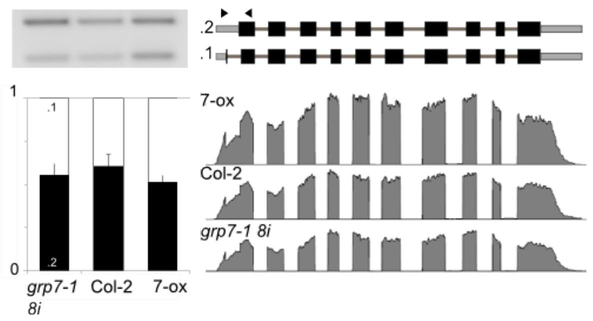

E AT3G17100 (*AIF3*)

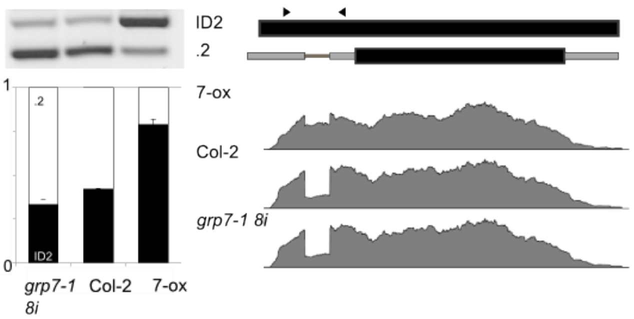

F AT1G33265 (*FAX4*)

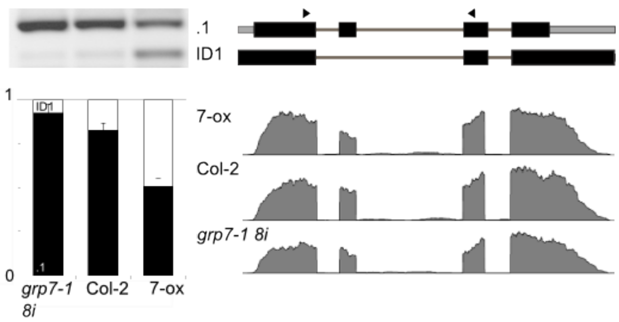

G AT1G76020 (*Thioredoxin superfamily protein*)

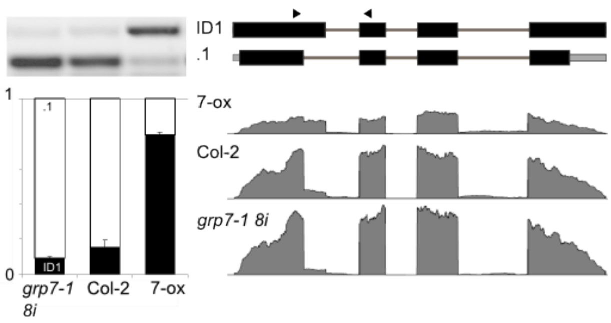

Figure S15 Validation of differential alternative splicing of *At*GRP7 targets at LL24

Each panel depicts a representative RT-PCR result of the alternative splicing event (top left), the gene model of the representative isoforms based on the atRTD annotation [5] (top right), a bar plot indicating the expected relative amount of the isoform calculated from the RNA-seq data (bottom left) and an RNA coverage plot visualizing the read coverage (bottom right).

At LL24 the intron retention of *TCH3* (A) in *grp7-1 8i* is not significantly different from the wild type. *FNR2* (B) shows enhanced intron retention in *At*GRP7-ox compared to the wild type. A GDSL-Like Lipase/Acylhydrolase superfamily protein (C) also retains intron 1 more often in *At*GRP7-ox than in the wild type. In contrast to LL36 the increase of the fully spliced isoform of Transducin/WD40 repeat-like superfamily protein (D) in *At*GRP7-ox does not meet our criteria of ΔPSI > 0.1 (0.095), but is statistically significant. Again, this is accompanied by elevated steady-state abundance in *At*GRP7-ox. *AIF3* (E) shows higher levels of intron retention in *At*GRP7-ox than in the wild type, whereas an exon of *FAX4* (F) is skipped more

often in *At*GRP7-ox than in the wild type. A thioredoxin superfamily protein (G) shows a differential usage of an alternative 5' splice site in *At*GRP7-ox.
Events were validated by RT-PCR in two independent biological replicates taken at LL24, shown are representative results from *grp7-1 8i*, Col-2 and *At*GRP7-ox (7-ox) samples (left to right). Gene models are shown for the relevant isoforms with thin bars representing the 5'UTR (left) and 3'UTR (right), thick bars denoting exons and lines depicting introns. Arrows indicate the position of the PCR primers. The bar plots represent the expected relative amount of the indicated isoform calculated from the RNA-seq data using SUPPA in three independent biological replicates. The RNA coverage plots visualize the read coverage for the respective samples at LL24. Information on the transcripts is taken from TAIR 10 and ARAPORT [6].

Supplemental references

1. Nettling M, Treutler H, Grau J, Keilwagen J, Posch S, Grosse I: **DiffLogo: a comparative visualization of sequence motifs.** *BMC Bioinformatics* 2015, **16:**387.
2. Schöning JC, Streitner C, Page DR, Hennig S, Uchida K, Wolf E, Furuya M, Staiger D: **Autoregulation of the circadian slave oscillator component *At*GRP7 and regulation of its targets is impaired by a single RNA recognition motif point mutation.** *Plant Journal* 2007, **52:**1119-1130.
3. Schüttpelz M, Schöning JC, Doose S, Neuweiler H, Peters E, Staiger D, Sauer M: **Changes of conformational dynamics of mRNA upon AtGRP7 binding studied by fluorescence correlation spectroscopy.** *Journal of the American Chemical Society* 2008, **130:**9507-9513.
4. Leder V, Lummer M, Tegeler K, Humpert F, Lewinski M, Schüttpelz M, Staiger D: **Mutational definition of binding requirements of an hnRNP-like protein in *Arabidopsis* using fluorescence correlation spectroscopy.** *Biochemical and Biophysical Research Communications* 2014, **453:**69-74.
5. Zhang R, Calixto CPG, Tzioutziou NA, James AB, Simpson CG, Guo W, Marquez Y, Kalyna M, Patro R, Eyras E, et al: **AtRTD – a comprehensive reference transcript dataset resource for accurate quantification of transcript-specific expression in Arabidopsis thaliana.** *New Phytologist* 2015, **208:**96–101.
6. The International Arabidopsis Informatics Consortium: **Taking the Next Step: Building an Arabidopsis Information Portal.** *The Plant Cell* 2012, **24:**2248-2256.
